# Preformulation Studies and Rational Design of an Ointment Containing a Postbiotic Metabolite of Procyanidins for Topical Use

**DOI:** 10.3390/pharmaceutics18060749

**Published:** 2026-06-18

**Authors:** Tomasz Todryk, Monika Budnicka, Lukasz Pajchel, Hanna Kierońska, Maciej Dawidowski, Krzysztof Adam Stępień, Joanna Giebułtowicz, Sebastian Granica, Joanna Kolmas, Jakub P. Piwowarski

**Affiliations:** 1 Department of Pharmaceutical Chemistry and Biomaterials, Faculty of Pharmacy, Medical University of Warsaw, Banacha 1 Street, 02-097 Warsaw, Poland; s086132@student.wum.edu.pl (T.T.); monika.budnicka@wum.edu.pl (M.B.); lukasz.pajchel@wum.edu.pl (L.P.); 2 Pikralida Sp. z.o.o, Uniwersytetu Poznańskiego 10 Street, 61-614 Poznan, Poland; h.kieronska@pikralida.eu; 3 Department of Drug Technology and Pharmaceutical Biotechnology, Medical University of Warsaw, Banacha 1 Str., 02-097 Warsaw, Poland; maciej.dawidowski@wum.edu.pl; 4 Department of Drug Chemistry, Faculty of Pharmacy, Medical University of Warsaw, Banacha 1 Street, 02-097 Warsaw, Poland; krzysztof.stepien@wum.edu.pl (K.A.S.); joanna.giebultowicz@wum.edu.pl (J.G.); 5 Department of Pharmaceutical Biology, Medical University of Warsaw, Banacha 1 Street, 02-097 Warsaw, Poland; sebastian.granica@wum.edu.pl; 6 Microbiota Lab, Department of Pharmaceutical Microbiology and Bioanalysis, Medical University of Warsaw, Banacha 1 Street, 02-097 Warsaw, Poland; jakub.piwowarski@wum.edu.pl

**Keywords:** atopic dermatitis, topical formulation, anti-inflammatory effect, gut microbiota-derived metabolites, flavanol, preformulation

## Abstract

**Background:** 5-(3′,4′-Dihydroxyphenyl)-γ-valerolactone (DHPV) is a postbiotic gut microbiota-derived flavanol metabolite with reported anti-inflammatory activity. Despite growing interest in its potential dermatological applications, its pharmaceutical properties and suitability for topical delivery have not been systematically investigated. This study aimed to perform the first comprehensive preformulation and formulation-oriented evaluation of DHPV and to develop stable topical ointment formulations suitable for further dermatological research. **Methods:** The physicochemical properties of DHPV were characterized using powder X-ray diffraction (PXRD), Fourier-transform infrared spectroscopy (FTIR), scanning electron microscopy (SEM), quantitative solubility assessment, and excipient compatibility studies. Based on the obtained preformulation data, two anhydrous ointment formulations containing DHPV were developed. The formulations were evaluated for homogeneity, rheological behavior, chemical stability under accelerated storage conditions, and in vitro drug release performance. **Results:** DHPV was identified as a crystalline compound with heterogeneous particle morphology and limited aqueous solubility. Quantitative solubility studies demonstrated the highest solubility in PEG 300 and glycol-based solvents. Compatibility testing revealed increased impurity formation in hydrophilic environments, whereas lipophilic excipients provided improved chemical stability. Both ointment formulations exhibited acceptable physical characteristics and maintained DHPV stability throughout accelerated storage. However, marked differences in release behavior were observed. The lipid–wax formulation showed significantly higher release rates, lower variability, and more reproducible release profiles than the petrolatum-based reference formulation, indicating more efficient diffusion of DHPV from the semisolid matrix. **Conclusions:** The physicochemical characteristics of DHPV strongly influence formulation design and performance. Anhydrous lipid-based systems provide a favorable environment for maintaining DHPV stability, while formulation composition significantly affects drug release. The developed lipid–wax formulation represents a promising platform for future skin permeation, pharmacodynamic, and efficacy studies.

## 1. Introduction

**Figure 1:**
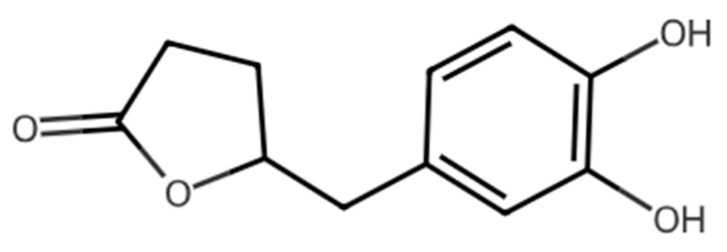
Chemical formula of 5-(3′,4′-dihydroxyphenyl)-γ-valerolactone (DHPV).

Atopic dermatitis (AD) is a chronic inflammatory skin disorder marked by pruritus, impaired epidermal barrier function, and increased susceptibility to microbial colonization [1,2]. The pathogenesis of AD is complex and involves genetic predisposition, immune dysregulation, changes in the skin microbiome, and environmental factors [2,3]. The stratum corneum in AD exhibits structural defects, often related to filaggrin deficiency [4,5]. These defects lead to increased transepidermal water loss (TEWL) and enhanced penetration of allergens and irritants. All these factors contribute to the perpetuation of inflammation and the chronicity of the disease [6]. Oxidative stress also appears to be a significant factor in the pathophysiology of AD [7]. Excessive production of reactive oxygen species (ROS) promotes lipid peroxidation and keratinocyte apoptosis, further compromising the epidermal barrier and enhancing inflammatory signaling [7,8]. Therefore, compounds with both anti-inflammatory and antioxidant effects appear particularly interesting in the topical treatment of atopic dermatitis. Standard therapies include emollients, topical glucocorticosteroids, calcineurin inhibitors (tacrolimus and pimecrolimus), and, in severe cases, systemic or biologic therapies (e.g., dupilumab and tralokinumab) [9,10,11]. However, long-term use of corticosteroids is associated with adverse effects such as skin atrophy, telangiectasia, and pigmentation disorders, and some patients demonstrate an inadequate response to available treatments [9]. Although nonsteroidal topical agents and biologic therapies have expanded the therapeutic options for AD, their use may be limited by tolerability issues, cost, or the need for long-term treatment, underscoring the continuing need for safe and well-tolerated topical preparations. In recent years, increasing attention has been paid to postbiotic metabolites—bioactive compounds produced by microbial metabolism of dietary components [12,13]. Among these, 5-(3′,4′-dihydroxyphenyl)-γ-valerolactone (DHPV) can be highlighted (see Figure 1). DHPV is a main gut microbiota-derived metabolite formed from flavan-3-ols such as catechins and procyanidins contained in various food and pharmaceutical plant products [14]. Recent studies have demonstrated its potent antioxidant and anti-inflammatory effects in vitro and in vivo [15,16,17].

DHPV has been shown to inhibit the production of proinflammatory cytokines and modulate key signaling pathways, including NF-κB and MAPK, while exhibiting greater chemical stability and bioavailability than its parent polyphenols, procyanidins [17,18]. DHPV is produced by gut microbiota from flavanol-rich foods and beverages, but its metabolism and conjugation limit its bioactivity in peripheral tissues [18,19,20,21]. Local administration to the site of inflammation may therefore represent a promising avenue for utilizing its anti-inflammatory and antioxidant properties while minimizing systemic exposure. It is worth mentioning that ointment formulations are particularly attractive in the context of atopic dermatitis due to their occlusive properties, ability to reduce TEWL, and favorable tolerance on damaged skin [20,21].

Topical administration appears particularly attractive for DHPV because atopic dermatitis is a localized inflammatory skin disorder and local drug delivery may provide therapeutic concentrations directly at the site of action while minimizing systemic exposure [22,23,24]. In addition, topical administration may reduce the risk of extensive metabolic transformation and improve local availability of DHPV within affected skin regions [23,24,25,26]. Therefore, the development of a stable topical formulation was considered a rational strategy for exploring the therapeutic potential of this microbiota-derived metabolite.

Given the catechol structure of DHPV, it poses significant formulation challenges, particularly regarding solubility, chemical stability, and compatibility with commonly used excipients. This may explain why, despite growing interest in the biological activity of flavanol metabolites, DHPV remains largely unexplored from a pharmaceutical and formulation perspective. In particular, there is currently no data regarding its detailed physicochemical properties, compatibility with excipients, or suitability for topical application. Therefore, the aim of this study was to perform a comprehensive preformulation characterization of DHPV and to rationally design an appropriate anhydrous topical formulation based on its physicochemical and stability constraints. The work focused on (i) physicochemical characterization of the active ingredient, (ii) systematic evaluation of DHPV solubility in solvents and excipients relevant to formulation, (iii) compatibility studies assessing chemical stability and impurity formation in binary DHPV–excipient mixtures, and (iv) development and comparative evaluation of DHPV-containing ointment formulations. The obtained results provide a rational basis for selecting an appropriate vehicle for topical administration of DHPV in the treatment of atopic dermatitis. The physicochemical characteristics of DHPV were considered particularly relevant for formulation development because properties such as solid-state structure, particle morphology, solubility profile, and chemical stability directly influence the selection of excipients, processing conditions, homogeneity of semisolid systems, and long-term product stability. Therefore, the present study aimed not only to characterize DHPV as an active pharmaceutical ingredient but also to establish a direct relationship between its physicochemical properties and the design of a suitable topical dosage form.

## 2. Materials and Methods

### 2.1. Reagents

1,2-propanediol and 1,3-propanediol were obtained from Sigma-Aldrich (St. Louis, MO, USA) and Ethanol 96%, glycerin, PEG 300, Polisorbat 80 and Polisorbat 60 from PolAura (Morąg, Poland). Formic acid and acetonitrile were purchased from Merck (Darmstadt, Germany). Petrolatum, Medium-chain-triglycerides, Paraffinum liquidum, Castor oil, Isopropyl Myristate, White wax, Cetostearyl Alcohol, Cetyl Palmitate 94%, and Sorbitan Monostearate (SPAN 60) were purchased from Merck (Darmstadt, Germany).

### 2.2. Active Pharmaceutical Ingredient

5-(3′,4′-Dihydroxyphenyl)-γ-valerolactone (DHPV) was synthesized at the Medical University of Warsaw at a purity exceeding 98%, as confirmed by HPLC and NMR analysis (see Supplementary Materials, Figure S1). The material was stored at 4 °C, protected from light, until it was used.

### 2.3. Physicochemical Characterization of DHPV

The surface morphology and particle characteristics of DHPV were examined using optical and scanning electron microscopy (Phenom Pharos G2, ThermoFisher Scientific, Waltham, MA, USA). In SEM experiments, samples were mounted on aluminium stubs, sputter-coated with a conductive gold layer, and analyzed at an accelerating voltage of 10 kV and magnification settings of 10,000–15,000. SEM and optical microscopy analysis were performed to assess particle morphology and surface features relevant to dispersion in semisolid formulations. Particle size estimates were based on qualitative assessment of SEM images and were not derived from statistical size distribution analysis.

The mid-infrared spectroscopy method (FTIR) was used to detect the functional groups and the chemical structure of the DHPV sample. The spectra were acquired on a Spectrum1000 spectrometer with 30 scans in the range of 4000–400 cm^−1^, with a resolution of 2 cm^−1^. The conventional transmission technique with a KBr tablet was used.

Powder X-ray diffraction analysis was performed to assess the solid-state properties of DHPV. Diffraction patterns were recorded using a Bruker D8 Discover diffractometer (Bruker, Madison, WI, USA) and collected over a 2θ range of 10° to 40°, utilizing Cu Kα radiation (λ = 1.54056 Å, 40 mA, 40 kV), with a step increment of 0.03. The Bragg–Brentano geometry was used.

#### 2.3.1. Solubility Studies

Qualitative Ph. Eur. solubility classification of DHPV was evaluated in purified water and selected solvents and excipients relevant to topical formulations, including ethanol (96%), 1,2-propanediol, 1,3-propanediol, glycerin, polyethylene glycol 300 (PEG 300), polysorbate 60, and polysorbate 80.

Preliminary equilibrium solubility of DHPV was determined using a shake-flask method. Excess DHPV was added to the tested solvents, and the suspensions were shaken at 25 ± 2 °C for 6 h. After equilibration, undissolved material was removed by filtration through a 0.45 μm membrane filter. The concentration of dissolved DHPV was quantified using a validated HPLC method. All measurements were performed in triplicate, and the results were expressed as % (*w*/*v*) solubility values.

##### HPLC Analysis

Quantitative analysis of DHPV was performed using a Shimadzu LC-2030C 3D Plus HPLC system equipped with a reversed-phase C_18_ column (CORTEX Shield RP_18_, 150 × 4.6 mm, 2.7 μm) and a diode array detector. The detection was performed at 280 nm.

Chromatographic separation was performed using gradient elution with mobile phase A consisting of water containing formic acid (1000:1, *v*/*v*) and mobile phase B consisting of acetonitrile. The gradient program was as follows: 0.01 min, 100% A; 30.0 min, 10% A; 34.0 min, 10% A; 34.1 min, 100% A; 40.0 min, 100% A. The flow rate was set at 1.2 mL/min, and the column temperature was maintained at 30 °C. The autosampler temperature was set at 20 °C. The injection volume was 5 µL. Detection was carried out using a diode array detector at 280 nm. The total run time was 40 min. Before sample analysis, method suitability was validated (see Supplementary Materials, Table S1 and Figures S2 and S3). The content of DHPV was calculated using the peak-area normalization method.

#### 2.3.2. Stability of DHPV Solutions

The short-term stability of DHPV solutions obtained during solubility studies was evaluated to support formulation development. Aqueous and selected non-aqueous solutions (ethanol, 1,2-propanediol, 1,3-propanediol, glycerin, PEG300, polysorbate 60 and polysorbate 80) of DHPV were stored in transparent glass containers under laboratory conditions without light protection and visually inspected over time for signs of physical or chemical instability, including color change. Quantitative chemical analysis of solutions was not performed at this stage because the purpose of the experiment was rapid excipient screening rather than kinetic characterization. Additionally, the effects of temperature and light on aqueous solutions were studied. The solutions were stored in the refrigerator (5 ± 1 °C) and at room temperature (25 ± 2 °C) in clear and amber bottles. Changes in appearance were used as a qualitative indicator of instability.

### 2.4. Compatibility Studies

Compatibility studies were conducted to evaluate the physicochemical stability of DHPV in the presence of excipients commonly used in topical ointment formulations. Fourteen binary mixtures consisting of DHPV and a single excipient were prepared (Table 1). In addition, the Active Pharmaceutical Ingredient (API) and selected excipients (Medium-Chain Triglycerides- MCT oil, 1,2-propanediol, 1,3-propanediol, glycerin, PEG300) were analyzed as individual control samples.

Excipients included hydrocarbons, oils, glycols, waxes, fatty alcohols, and emulsifiers. Binary mixtures were prepared at predefined weight ratios representative of their intended use in the final formulation (see Table 1). The solids were heated above the melting point, and DHPV was added to the melted components. All samples were thoroughly mixed, transferred into identical 10 mL glass containers sealed with aluminium caps, and stored under the following conditions: accelerated conditions (40 ± 2 °C) and room temperature (25 ± 2 °C) with uncontrolled humidity in both cases. Samples were visually inspected and analyzed after 14 and 28 days of storage. Time-zero chemical analysis was not performed as the focus was on comparative impurity evolution rather than absolute degradation kinetics. However, the solutions were compared to the DHPV control sample. Quantitative analysis of DHPV and impurities was performed using HPLC (method described in Section 2.3.1). The content of related substances and impurities was calculated using the peak-area normalization method. The method provided adequate resolution of the chromatographic peaks of DHPV and its impurities. For early-stage excipient screening, a total impurity level of 1.0% was applied as an internal ranking criterion to compare the relative impact of excipients on DHPV stability. This threshold was used solely to support formulation selection.

### 2.5. Preparation of Ointment Formulations Containing DHPV

Two anhydrous ointment formulations containing DHPV were developed based on the outcomes of preformulation and compatibility studies. In both cases, DHPV was used without any preliminary treatment such as micronization or pre-dissolution.

All selected excipients were weighed accurately and combined in a vessel. The formulation components were heated to their respective melting temperatures (60 °C and 63 °C for formulations 1 and 2, respectively) under controlled conditions until a homogeneous molten phase was obtained. DHPV was then incorporated into the molten base.

The resulting mixtures were subjected to a first homogenization step at 60 °C using a high-shear homogenizer operating at 15,000 rpm for 5–6 min to decrease the size of API’s particles and ensure uniform API dispersion. Following homogenization, the formulations were cooled at ambient temperature while continuously mixing until the temperature decreased to approximately 30 °C.

A second homogenization step was subsequently performed at 30 °C at a reduced speed of 12,000 rpm for 2 min to improve homogeneity and to minimize the risk of uneven ointment base solidification and oil phase separation. After complete cooling and solidification, the ointments were filled into aluminium tubes equipped with an internal membrane and screw cap. The prepared ointments were stored at controlled room temperature and protected from light before further evaluation. For each formulation, four API concentrations (0.5%, 1.0%, 2.0%, and 5.0% *w*/*w*) were prepared, as well as a formulation without API (placebo).

#### 2.5.1. Viscosity Tests

For each formulation, two DHPV concentrations (0.5% and 5.0% *w*/*w*) were selected to assess the influence of drug loading on rheological and stability properties. Dynamic viscosity measurements were performed at 25 °C using a rotational rheometer (RHEOPLUS/32 V3.62, Anton Paar, Graz, Austria) equipped with a cone-and-plate geometry (CP25, gap 0.049 mm). Viscosity was recorded at the constant temperature of 25 °C, as a function of shear rate, and comparative values were extracted at 50.4 s^−1^. Comparative values are reported at a representative shear rate relevant to topical application. Detailed rheological modeling was beyond the scope.

#### 2.5.2. Stability of the Formulations

Stability studies were conducted on formulations containing 0.5% and 5.0% API, as well as on the corresponding placebo formulations. Samples were stored in aluminum tubes under ICH-recommended conditions: 25 °C/60% RH, 30 °C/65% RH, and 40 °C/75% RH. At predefined time points (initial, 28 days, 3 months), samples were evaluated for appearance, impurities, DHPV content, and microscopic structure, depending on study stage and formulation type.

DHPV content and impurities were quantified using a validated reversed-phase HPLC method with UV detection (parameters described above). Samples were taken from the top and bottom portions of tubes to assess content uniformity. Total impurities and individual impurity levels were calculated based on peak area normalization.

#### 2.5.3. In Vitro Release Test (IVRT)

The in vitro drug release test was conducted in a USP apparatus 2 with immersion cell model B (USP<1724>). The immersion cell had an exposed area of 1.5 cm^2^. Saline containing phosphate buffer (pH 7.4, 10 mM), pre-heated to 32 °C was used as the dissolution medium. A hydrophobic polyethersulfone membrane filter (25 mm diameter, 0.45 μm pore size; cat. no. PSMDC-PS25-045-0100, Dissolution Accessories, Oosterhout, The Netherlands) was used as the barrier membrane. Before the experiment, membrane inertness toward DHPV was evaluated to confirm the absence of nonspecific adsorption or interactions that could affect DHPV recovery and release kinetics. The bottom screw of the immersion cell reservoir was tightened with the adjustment tool to hold approximately 0.550 g of ointment. The formulation was spread uniformly, and the surface leveled. The assembled immersion cell, with membrane facing up, was placed at the bottom of the dissolution vessel. The paddle height was set at 1.0 cm above the cell surface and rotated at 100 rpm. The temperature of each cell was maintained at 32.0 °C. Aliquots (1 mL) were collected at predetermined intervals up to six hours. The in vitro drug release rate was determined by fitting drug release data to the Higuchi model.

#### 2.5.4. LC–MS/MS Analysis

Quantitative analysis of DHPV was performed using a liquid chromatography–tandem mass spectrometry system consisting of a Shimadzu LC40D XR system coupled with a Shimadzu LCMS-8050RX triple quadrupole mass spectrometer (Shimadzu Corporation, Kyoto, Japan) equipped with an electrospray ionization source. Chromatographic separation was achieved using BionaCore C18 UFPLC column (4.6 mm × 100 mm, 2.7 µm) maintained at 40 °C. The mobile phase consisted of solvent A (water with 0.1% formic acid, *v*/*v*) and solvent B (acetonitrile with 0.1% formic acid, *v*/*v*). Gradient elution was applied at a flow rate of 0.750 mL/min. The injection volume was 5 µL. Mass spectrometric detection was performed in positive ionization mode using multiple reaction monitoring. The precursor-to-product ion transition monitored was *m*/*z* 209.00 → 131.00 (collision energy, CE = 20 V) for DHPV and *m*/*z* 152.00 → 110.10 (CE = 18 V) for acetaminophen (internal standard). The following source parameters were applied: interface voltage 1.0 kV, desolvation line temperature 250 °C, heat block temperature 400 °C, nebulizing gas flow 3 L/min, heating gas flow 10 L/min, and drying gas flow 10 L/min. Data acquisition and processing were carried out using LabSolutions software v. 5.135SP1 (Shimadzu Corporation, Kyoto, Japan). 

## 3. Results

### 3.1. Characterization of the Synthesized DHPV

Preformulation studies assessed the physicochemical properties of the synthesized DHPV and its suitability for incorporation into semi-solid dosage forms intended for use on inflamed skin. This study intentionally focuses on preformulation and physicochemical stability as a prerequisite for further biopharmaceutical evaluation. In this context, particular attention was paid to solubility, compatibility, and stability of the API.

**Figure 2:**
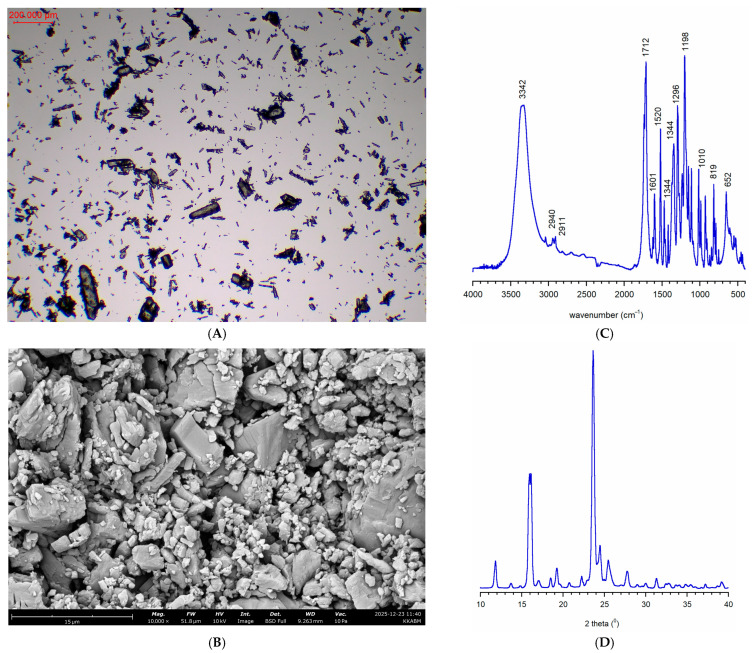
Physicochemical studies of the DHPV samples: (**A**) Optical microscopy image (scale bar = 200 μm) revealing heterogeneous particle size distribution; (**B**) SEM micrograph (magnification 10,000×) showing irregular, angular crystalline particles; (**C**) FTIR and (**D**) PXRD studies.

DHPV was obtained for analysis as an off-white powder with a bulk density of 0.482 g/cm^3^. Preliminary optical microscopic and SEM analysis (Figure 2A and Figure 2B, respectively) showed that the DHPV sample was a crystalline substance dominated by small particles measuring 10–100 μm in length and up to 15 μm in width. Large columnar particles measuring 160–610 μm in length and 40–140 μm in width were also present in the sample (see Figure 2).

Optical microscopy (Figure 2A) revealed that DHPV consisted of irregularly shaped crystals with a heterogeneous size distribution. Most crystals were slightly elongated and showed a weak tendency to aggregate. No spherical or amorphous grains were observed, suggesting a fully crystalline material. SEM images (Figure 2B) confirmed the crystalline nature of DHPV, revealing angular, faceted particles with rough surfaces and sharp edges. This morphology reveals a sharp-edged structure potentially prone to fracture during processing, as commonly reported for angular crystalline particles [22]. It should be noted that particle size assessment was based on microscopic observations and therefore provided only qualitative information regarding particle heterogeneity. Quantitative particle size distribution analysis should be addressed in future investigations.

The FTIR spectrum of DHPV showed an intense and broad band at approximately 3342 cm^−1^, corresponding to the O–H stretching vibrations of the phenolic groups (Figure 2C). The bands at 2940 and 2911 cm^−1^ can be attributed to the C–H stretching vibrations of the groups. A very intense band at 1712 cm^−1^ indicates the presence of a carbonyl group (C=O) bonded to the lactone moiety. Additional characteristic bands were observed at 1601 and 1520 cm^−1^, corresponding to aromatic C=C stretching vibrations, while bands in the 1344–1010 cm^−1^ range may originate from in-plane C–O stretching vibrations and aromatic C–H bending vibrations [27,28]. Overall, the spectrum is consistent with the expected chemical structure of DHPV.

The PXRD pattern (Figure 2D) confirmed the crystalline nature of DHPV. The reflections in the pattern are intense and narrow, indicating an ordered structure. The main reflections were observed at approximately 2θ ≈ 15°, 17°, 21°, 23°, and 25°, with the most intense reflection at approximately 23°. The lack of evidence of halo broadening may indicate no detectable amorphous phase in the tested sample.

From a formulation perspective, these observations provided several important design considerations. First, the fully crystalline nature of DHPV and the absence of detectable amorphous content suggested that dissolution of the API within semisolid vehicles would be limited, favoring suspension-type formulations rather than systems relying on complete solubilization [29]. Second, the heterogeneous particle size distribution indicated that efficient homogenization would be required to ensure acceptable content uniformity and reproducible dispersion throughout the ointment matrix. Finally, the angular crystalline morphology suggested that particle size reduction during processing could potentially improve dispersion and formulation homogeneity. These findings were subsequently taken into account during excipient selection and ointment manufacturing.

### 3.2. Solubility and Solution Stability

Following the characterization of the solid-state properties of DHPV, equilibrium solubility studies were performed to determine whether the API could be incorporated into topical formulations as a dissolved substance or whether a suspension-based approach would be required (Table 2). Quantitative shake-flask experiments followed by HPLC analysis demonstrated substantial differences in the solubilizing capacity of the investigated solvents and excipients.

The highest solubility of DHPV was observed in PEG 300 (18.09% *w*/*v*), followed by 1,3-propanediol (16.50% *w*/*v*), 1,2-propanediol (13.87% *w*/*v*), and ethanol (9.84% *w*/*v*). In these media, DHPV formed homogeneous and visually clear solutions at equilibrium. Moderate solubility was observed in Polysorbate 60 and Polysorbate 80, reaching 2.40% *w*/*v* and 2.03% *w*/*v*, respectively. The lowest solubility was found in glycerin (0.87% *w*/*v*) and water (0.65% *w*/*v*), indicating limited suitability of these vehicles for achieving therapeutically relevant concentrations of dissolved DHPV.

Overall, the results demonstrate a clear preference for DHPV for glycol-based solvents and PEG 300, while aqueous systems exhibit substantially lower solubilization capacity.

For preliminary stability assessment, the saturated solutions obtained during the solubility studies were stored at room temperature without protection from light and monitored visually. The DHPV solutions in glycols, PEG 300, glycerin, and polysorbates showed no changes in appearance. Unfortunately, the aqueous solution showed a color change from colorless to pink the next day. This color became more intense with each passing day, likely due to autooxidation of DHPV, although degradation products were not structurally characterized in this study [30,31]. The ethanol solution exhibited a similar behavior, but the rate of color change was slower. Therefore, the aqueous and ethanol solutions were considered chemically unstable; however, these observations are qualitative and should be interpreted with caution in the absence of quantitative analytical confirmation. The obtained data constitute a preliminary assessment of the technological capabilities and stability of the active substance and formed the basis for further formulation development. These findings are consistent with the known chemical behavior of catechol-containing compounds, which are prone to autooxidation in aqueous, oxygen-rich environments [32]. The observed color change likely reflects the formation of o-quinone structures and subsequent polymerization products, which have been widely reported for structurally related phenolic systems [33]. From a formulation perspective, these results clearly indicate that aqueous systems are unsuitable for DHPV delivery and further justify the selection of anhydrous vehicles.

In addition to supporting the exclusion of aqueous vehicles, the results of the solubility and solution stability screening also guided the selection of excipients for subsequent compatibility studies. Although DHPV exhibited high equilibrium solubility in glycols and PEG 300, the simultaneous observation of instability indicated that solubility alone could not be used as the primary criterion for formulation design. Instead, a balance between solubilization capacity and chemical stability had to be achieved. This finding proved critical for the subsequent development of anhydrous lipid-based ointment systems, where chemical stability was prioritized over complete API solubilization. A detailed analysis of DHPV degradation will be the subject of our further studies.

### 3.3. Compatibility Studies

To assess the compatibility of DHPV with selected excipients used in topical drug formulations, the appearance assessment and HPLC content measurements of DHPV in binary mixtures (DHPV + excipient) were performed under controlled conditions for 14 and 28 days at room temperature and 40 °C (see Table 3).

No changes in the appearance of either DHPV or its mixtures with excipients were observed over the 14 days. However, after 28 days of storage, slight changes in the appearance of DHPV mixtures with MCT oil, paraffin oil, PEG 300 and castor oil were observed. These changes (a color change from white to cream, darkening of the solution or thickening of the suspension) were visible regardless of storage temperature. However, at 40 °C, slight changes in the appearance of mixtures with cetostearyl alcohol, propylene glycol, and sorbitan stearate were observed.

Table 3 summarizes content of impurities in all 14 mixtures of DHPV and chosen excipients, which were acquired by HPLC analysis. For formulation screening, a provisional acceptance criterion of ≤1.0% total impurities was applied, which is consistent with commonly used limits for early-stage topical drug development [34]. What is important, impurity 1, identified as 5-(3,4-dihydroxyphenyl)pentanoic acid, was consistently detected in the DHPV control samples and therefore represents an intrinsic DHPV-related impurity rather than a product of DHPV–excipient incompatibility (Supplementary Materials, Figures S4 and S5). After 28 days in 40 ± 2 °C conditions, among all 14 mixtures, only in 4 of them (E5–E8): mixture of DHPV with 1,2-propanediol, 1,3-propanediol, PEG 300 and glycerin, the content of total impurities exceeded 0.5%. The mixture with PEG 300 exhibited a value of total impurities over 1.0% and stands at 1.1%. For the mixtures mentioned above, additional tests were performed to determine if peaks of potential impurities do not result from the excipient alone but rather from incompatibility between API and excipient (chromatograms of excipients stored in 40 ± 2 °C conditions for 28 days were compared to mixtures stored at room temperature for 28 days) (see Table S2 in Supplementary Materials).

Comparative analysis of control excipient samples confirmed that the observed impurity peaks resulted from API–excipient interactions rather than solely from excipient degradation. The increased impurity formation observed in glycol-based solvents, glycerin, and PEG 300 may be related to the presence of the catechol moiety in the DHPV structure, which is known to be susceptible to oxidative degradation [31,32]. Catechol-containing compounds can undergo autoxidation to reactive o-quinone intermediates, particularly in polar environments, leading to the formation of secondary degradation products. Consistent with this behavior, hydrophilic excipients were generally associated with higher impurity levels, whereas lipophilic excipients exhibited lower impurity formation and better preservation of DHPV stability under accelerated storage conditions. Although the exact degradation and stabilization mechanisms were not investigated in the present study, these findings highlight the importance of considering both solubilization properties and chemical stability during excipient selection and support the use of anhydrous lipid-based systems for DHPV formulation.

The compatibility results also provided a rationale for formulation selection. While hydrophilic excipients offered superior DHPV solubilization, they simultaneously promoted impurity formation and chemical instability. In contrast, lipophilic excipients provided a chemically protective environment despite the limited solubility of the API. Consequently, formulation development prioritized chemical stability over complete API solubilization and focused on the design of suspension-type anhydrous ointment systems.

### 3.4. Development of Ointment Formulations Containing DHPV

Based on the preformulation studies, including solid-state characterization, particle morphology assessment, solubility screening, and compatibility testing, two anhydrous ointment formulations containing DHPV were developed.

The formulation strategy was directly influenced by the physicochemical characteristics of DHPV. The limited aqueous solubility, crystalline nature, particle size heterogeneity, and susceptibility to degradation in hydrophilic environments collectively supported the development of suspension-based anhydrous systems composed predominantly of lipophilic excipients. The compositions of the prepared formulations are summarized in Table 4.

**Figure 3:**
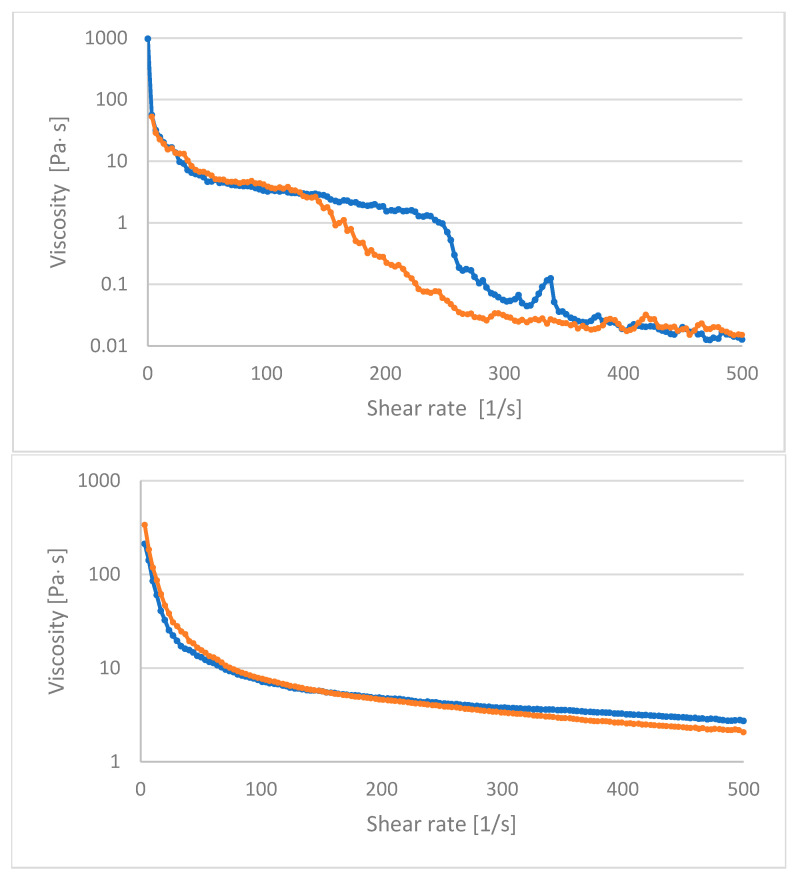
Viscosity vs. shear rate curves for Formulation 1 and 2 (up and bottom, respectively). Blue and red curves: DHPV concentrations of 0.5% and 5.0%, respectively.

Formulation 1 (F1) was developed as a conventional reference ointment based primarily on white petroleum and cetostearyl alcohol, with DHPV incorporated at concentrations ranging from 0.5% to 5.0% (*w*/*w*). This composition was selected due to its strong occlusive properties, widespread dermatological use, and demonstrated compatibility of DHPV with hydrocarbon-based excipients [35,36]. The resulting formulation exhibited a uniform semi-solid consistency and acceptable macroscopic appearance across all tested API concentrations. Rheological analysis revealed pseudoplastic (shear-thinning) behavior for both tested concentrations, with dynamic viscosity values of 13.0 Pa·s and 15.5 Pa·s at a shear rate of 50.4 s^−1^ and a constant temperature of 25 °C, for the 0.5% and 5.0% formulations, respectively (Figure 3). The increase in the viscosity with increasing API concentration suggests a slight strengthening of the internal structure of the ointment matrix [37]. Accelerated stability studies conducted at 40 ± 2 °C showed that the measured DHPV content remained close to the nominal value, indicating limited chemical degradation of the API (Table S3, Supplementary Materials). However, increased variability in the API content was observed at elevated temperatures, particularly at higher drug loadings. This effect may be associated with limited API mobility and potential micro-scale heterogeneity within the highly viscous petrolatum matrix, leading to localized concentration heterogeneity (see Supplementary Materials, Figure S6). Although total impurity levels remained within acceptable limits, the increased relative standard deviation (RSD) suggested that this formulation may be sensitive to processing and storage conditions. Overall, formulation 1 provided a chemically protective environment for DHPV but exhibited limitations in compositional homogeneity under stress conditions.

The observed variability in API content at elevated temperatures may also be related to the limited solubility of DHPV in the petrolatum matrix, resulting in a suspension-type system. Under such conditions, restricted molecular diffusion and potential sedimentation or aggregation of crystalline particles may contribute to local concentration gradients. This effect is particularly relevant for highly viscous systems, where insufficient internal mobility can impair content uniformity despite apparent macroscopic homogeneity.

Formulation 2 (F2) was designed as a lipid-based system composed exclusively of medium-chain triglycerides and white wax. This composition aimed to improve API dispersion, enhance formulation homogeneity, and maintain chemical stability while preserving the advantages of an anhydrous ointment base [38,39,40]. Rheological measurements demonstrated significantly lower viscosity values compared with Formulation 1, amounting to 4.62 Pa·s and 6.34 Pa·s at 50.4 s^−1^ and a constant temperature of 25 °C, for the 0.5% and 5.0% formulations, respectively, while retaining pseudoplastic flow behavior. These results indicated a less densely structured matrix, which may translate into improved spread ability and patient acceptability. Across the entire tested concentration range (0.5–5.0% *w*/*w*), Formulation 2 demonstrated high physical homogeneity and reproducibility, with API contents consistently close to 100% and low RSD values. No evidence of phase separation or crystallization was observed during storage. Under accelerated conditions, total impurity levels remained low even at the highest API concentration. Compared with the petrolatum-based system, the lipid–wax formulation exhibited greater resistance to impurity formation, suggesting a more favorable microenvironment for the chemically labile catechol moiety of DHPV.

The improved performance of this formulation may be attributed to the more favorable balance between viscosity and molecular mobility within the lipid–wax matrix although this mechanism was not directly investigated in the present study. Compared to the petrolatum-based system, the lower viscosity likely facilitates more uniform API distribution and reduces the risk of localized supersaturation or crystallization. Additionally, MCT oil may enhance wetting of the DHPV particles, promoting better dispersion and contributing to the observed formulation homogeneity. These properties are particularly advantageous for topical applications, where consistent dosing and spreadability are critical.

The lower viscosity of the lipid–wax formulation (F2) may also facilitate improved drug diffusion within the matrix, which could potentially translate into enhanced release characteristics, although this requires further investigation.

From a broader perspective, the present study highlights the formulation challenges associated with catechol-containing bioactive compounds and underscores the critical role of excipient selection in mitigating oxidative instability. While DHPV demonstrates promising pharmacological activity, its successful translation into a topical dosage form requires careful control of the microenvironment to prevent degradation. The results obtained here may therefore be applicable not only to DHPV but also to other microbiota-derived phenolic metabolites with similar structural features.

**Figure 4:**
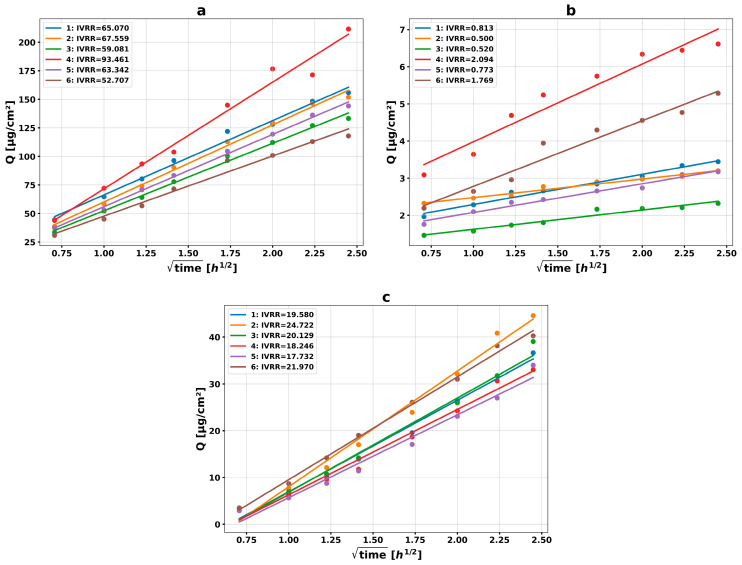
In vitro release test profiles of DHPV from 2% ointment formulations 2 (**a**) and 1 (**b**), as well as the 0.5% formulation 1 (**c**). For the 0.5% F1 formulation, drug release remained below the lower limit of quantification (LLOQ) of the analytical method throughout the experiment.

The in vitro release studies demonstrated substantial differences in the release behavior of the two 2% DHPV ointment formulations. In both cases, the cumulative amount of released compound increased proportionally to the square root of time, indicating diffusion-controlled release kinetics consistent with the Higuchi model, which is characteristic of semisolid topical dosage forms (see Figure 4) [41].

The formulation 2 exhibited markedly higher release rates compared to the formulation 1. The mean in vitro release rate (IVRR) for F2 was 67 ± 15 µg/cm^2^/h^1/2^, with a coefficient of variation (CV) of 21%, indicating relatively good reproducibility between replicates. Individual IVRR values ranged from 52.7 to 93.5 µg/cm^2^/h^1/2^. Moreover, all release profiles demonstrated high linearity, with determination coefficients (R^2^) between 0.979 and 0.997, confirming stable diffusion-driven release kinetics. The cumulative amount released at the final sampling (6 h) point reached approximately 118–212 µg/cm^2^, depending on the replicate. Such findings suggest that formulation promoted efficient diffusion of DHPV from the ointment base.

In contrast, the F1 formulation showed substantially lower release efficiency and markedly higher variability. The mean IVRR was 1.08 ± 0.69 µg/cm^2^/h^1/2^, with a high CV value of 64%, indicating poor reproducibility of the release process. Individual IVRR values varied considerably, ranging from 0.500 to 2.09 µg/cm^2^/h^1/2^. Lower linearity of the release profiles was also observed for this formulation, with R^2^ values between 0.943 and 0.978. The cumulative amounts released during the experiment remained low, reaching only approximately 2.3–6.6 µg/cm^2^ at the final time point (6 h). These results may indicate that the vehicle composition restricted the mobility and diffusion of DHPV within the semisolid matrix, thereby limiting drug release. 

Additional IVRT studies were also performed for formulations containing a lower concentration of DHPV (0.5%). A similar release trend was observed as for the 2% formulations. The ointment based on white petrolatum demonstrated substantially lower release efficiency compared to the wax-containing formulation. In particular, the petrolatum-based formulation did not reach the lowest limit of quantitation of the analytical method (IVRR < 0.33 µg/cm^2^/h^1/2^).

In contrast, the wax-containing formulation exhibited markedly higher release rates and good reproducibility of the release profiles. The mean IVRR was 20.4 ± 2.6 µg/cm^2^/h^1/2^, with a coefficient of variation as low as 13% and R^2^ values ranging from 0.977 to 0.996. Notably, despite a four-fold reduction in DHPV concentration compared with the 2% formulation, the release rate decreased by only approximately three-fold. This observation suggests that not all of the incorporated API in 2% formulation may be readily available for release from the semisolid matrix. However, considering the variability of the measurements, this effect should be interpreted with caution. From a formulation perspective, the results indicate that the wax-containing formulation maintains favorable diffusion characteristics even at low drug concentration, supporting efficient drug availability and release.

Previous studies have shown that rheological properties of semisolid formulations strongly influence both release rate and reproducibility in IVRT studies [42,43]. 

Overall, F2 demonstrated significantly superior release performance compared to F1, characterized by higher IVRR values, greater cumulative drug release, and lower inter-sample variability. These findings confirm that formulation composition plays a critical role in determining the release characteristics of DHPV and suggest that F2 provides more efficient and reproducible drug delivery under the applied IVRT conditions. 

## 4. Conclusions

This study represents the first comprehensive evaluation of preformulations and formulations of DHPV, a gut microbiota-derived flavanol metabolite with previously reported anti-inflammatory and antioxidant activity. Detailed physicochemical characterization provided essential information for formulation design and demonstrated that the formulation behavior of DHPV is strongly influenced by its solid-state properties, solubility profile, and chemical stability. The crystalline nature of the API, heterogeneous particle morphology, limited aqueous solubility, and susceptibility to degradation in hydrophilic environments collectively indicated that conventional aqueous or solvent-rich topical systems would be unsuitable. These physicochemical findings directly guided excipient selection and supported the development of anhydrous suspension-type ointment formulations. Two anhydrous ointment formulations were successfully developed and evaluated. Compared to the conventional petrolatum-based reference system, the lipid–wax formulation, consisting of MCT oleum and white wax, demonstrated improved DHPV homogeneity, physical stability, and consistently lower impurity formation under accelerated storage conditions.

Our results enable future studies on dermal penetration, pharmacodynamic efficacy, and clinical effectiveness in atopic dermatitis. We demonstrated that the chemical instability of catechol-containing DHPV could be mitigated through rational excipient selection, yielding a stable, homogeneous, anhydrous ointment suitable for topical application. A limitation of the present study is the lack of skin permeation and skin retention studies, which are required to determine whether the released DHPV can effectively penetrate the target skin layers. However, the present work was intentionally designed as a preformulation- and stability-focused study, providing a rational basis for subsequent biopharmaceutical investigations. Additional rheological studies, including viscoelastic and thixotropic properties, will be required to fully characterize the performance of the developed formulations. Future research will also be focused on biological tests in vitro and in vivo.

Contraindications for anhydrous ointments vary depending on the specific components but generally relate primarily to individual hypersensitivity. In the future, it will be appropriate to evaluate contraindications and precautions when preparing these ointments to avoid adverse reactions (hypersensitivity/allergy).

## Figures and Tables

**Table 1:** Selected excipients used in compatibility studies.

	Excipient	Function	DHPV: Excipient (*w*/*w*)
**E1**	Petrolatum	components of the ointment base	1:10
**E2**	Medium-chain-triglycerides		1:10
**E3**	Paraffinum liquidum		1:10
**E4**	Castor oil		1:10
**E5**	1,2-Propanediol	Solvent, penetration enhancer, moisturizing agent, preservative enhancer	1:5
**E6**	1,3-Propanediol		1:5
**E7**	PEG 300	Solvent, moisturizing agent	1:5
**E8**	Glycerin		1:5
**E9**	Isopropyl Myristate	Emollient, penetration enhancer	1:1
**E10**	White wax	Component of the ointment base, rheology modifier (thickening agent)	1:1
**E11**	Cetostearyl Alcohol	Emulsion stabilizer, viscosity enhancer	1:1
**E12**	Cetyl Palmitate 94%	Emollient, stabilizer	1:1
**E13**	Sorbitan Monostearate (SPAN 60)	W/O type emulsifier, emulsion stabilizer	1:1
**E14**	Polysorbate 60	O/W type emulsifier	1:1

**Table 2:** Equilibrium solubility (% *w*/*v*) of DHPV in water, solvents, and excipients relevant to topical formulation development.

	Solvent	Equilibrium Solubility (% *w*/*v*)	Solubility Class (Ph. Eur.)
1	Water	0.65%	Slightly soluble
2	Ethanol 96%	9.84%	Soluble
3	1,2-propanediol	13.87%	Freely soluble
4	1,3-propanediol	16.50%	Freely soluble
5	Glycerin	0.87%	Slightly soluble
6	PEG 300	18.09%	Freely soluble
7	Polysorbate 80	2.03%	Sparingly soluble
8	Polysorbate 60	2.40%	Sparingly soluble

**Table 3:** Impurity formation in binary mixtures of DHPV and selected excipients after storage under accelerated conditions (40 °C).

Name	Impurity 1 (wt.%)		Total Impurities (wt.%)	
	Day 14	Day 28	Day 14	Day 28
DHPV	0.185	0.201	0.230	0.250
E1	0.189	0.194	0.240	0.240
E2	0.194	0.188	0.370	0.440
E3	0.207	0.195	0.260	0.240
E4	0.198	0.201	0.240	0.300
E5	0.211	0.213	0.580	0.710
E6	0.211	0.212	0.420	0.570
E7	0.218	0.227	0.690	1.100
E8	0.206	0.201	0.410	0.570
E9	0.181	0.230	0.198	0.290
E10	0.199	0.213	0.250	0.310
E11	0.204	0.207	0.250	0.250
E12	0.203	0.213	0.250	0.260
E13	0.185	0.180	0.230	0.180
E14	0.203	0.198	0.250	0.250

**Table 4:** Composition of simple ointment bases.

Formulation 1	Content (%)	Formulation 2	Content (%)
DHPV	0.5 1.0 2.0 5.0	DHPV	0.5 1.0 2.0 5.0
Cetostearyl alcohol	15	White wax	30
Cetyl palmitate	2.0	MCT oleum	up to 100%
White petroleum	up to 100%		

## Data Availability

The original contributions presented in this study are included in the article. Further inquiries can be directed to the corresponding authors.

## Supplementary Materials

The following supporting information can be downloaded at: https://www.mdpi.com/article/10.3390/pharmaceutics18060749/s1. Figure S1. ^1^H NMR of the sample of the DHPV (5-[(3,4-dihydroxyphenyl)methyl]oxolan-2-one) used in this study. ^1^H NMR (400 MHz, CD_3_OD) δ 8.68 [br s, 2 × OH, ~0H (nominally 2H; exchanged into deuterium], 6.69 (br d, *J* = 10.6 Hz, 1H, C^5^H) overlapping 6.69 (br s, 1H, C^2^H), 6.55 (dd, *J* = 8.0, 2.1 Hz, 1H, C^6^H), {4.74–4.66 (m, 1H, CHO; [4.70 (pseudo-p, *J* = 6.3 Hz, 1H)]}, 2.86 (dd, *J* = 14.0, 6.2 Hz, 1H), 2.77 (dd, *J* = 14.0, 6.1 Hz, 1H), 2.52–2.41 (m, 1H), 2.32 (ddd, *J* = 17.7, 9.4, 4.8 Hz, 1H), 2.26–2.17 (m, 1H), 1.99–1.88 (m, 1H); Figure S2. (a–d)—representative chromatograms from validated HPLC method; (a) The example of blank chromatogram; (b) The example of Sensitivity test solution—Related substances test chromatogram; (c) The example of Standard solution—Assay test chromatogram; (d) The example of sample solution chromatogram; Figure S3. UV spectra of the DHPV (standard sample, concentration 0.4 mg/mL); Figure S4. Main impurity (Impurity 1) ^1^H NMR spectrum; Figure S5. HPLC chromatogram of the Impurity 1; Figure S6. Representative images taken under optical microscopy for formulations 1 and 2; Table S1. Validation data for the HPLC method; Table S2. Compatibility of DHPV with selected excipients was evaluated under accelerated conditions (40 ± 2°C). Impurity profiles are expressed as percentage content (HPLC results). Table S3. Representative data on the stability of formulations 1 and 2. (Packaging: aluminium tubes with membrane and screw cap (19 mm × 75 mm), approx. 8 g).

## Abbreviations

AD	Atopic Dermatitis
API	Active Pharmaceutical Ingredient
CV	coefficient of variation
DHPV	5-(3′,4′-Dihydroxyphenyl)-γ-valerolactone
FTIR	Fourier Transform Infrared Spectroscopy
HPLC	High-Performance Liquid Chromatography
IVRR	In vitro release rate
MAPK	Mitogen-Activated Protein Kinase
MCT	Medium-Chain Triglycerides
NF-κB	Nuclear Factor kappa B
NMR	Nuclear Magnetic Resonance
PEG	Polyethylene Glycol
Ph. Eur.	European Pharmacopoeia
PXRD	Powder X-ray Diffraction
ROS	Reactive Oxygen Species
RSD	Relative Standard Deviation
SEM	Scanning Electron Microscopy
TEWL	Transepidermal Water Loss
W/O	Water-in-Oil
O/W	Oil-in-Water
ICH	International Council for Harmonisation
RH	Relative Humidity
