# Supplementary material for: Preformulation Studies and Rational Design of an Ointment Containing a Postbiotic Metabolite of Procyanidins for Topical Use
Source: Pharmaceutics. 2026 Jun 18;18(6):749. doi: 10.3390/pharmaceutics18060749 (PMC13307394; doi:10.3390/pharmaceutics18060749)
Supplement: Supplementary file 1 [file pharmaceutics-18-00749-s001.zip › pharmaceutics-4310705-supplementary.pdf]

Supplementary Materials to:

Preformulation Studies and Rational Design of an Ointment Containing a Postbiotic Metabolite of Procyanidins for Topical Use

Tomasz Todryk[1], Monika Budnicka[1], Łukasz Pajchel[1], Hanna Kierońska[2], Maciej Dawidowski[3], Krzysztof Adam Stępień[4], Joanna Giebułtowicz[4], Sebastian Granica[5], Joanna Kolmas[1*],Jakub P. Piwowarski[6]

[1] Department of Pharmaceutical Chemistry and Biomaterials, Faculty of Pharmacy, Medical University of Warsaw, Banacha 1 str., 02-097 Warsaw, Poland

[2]Pikralida s. z.o.o, Uniwersytetu Poznańskiego 10 str., 61-614 Poznań, Poland

[3] Department of Drug Technology and Pharmaceutical Biotechnology, Medical University of Warsaw, Banacha 1 Str., 02-097 Warszawa, Poland.

[4] Department of Drug Chemistry, Faculty of Pharmacy, Medical University of Warsaw, Banacha 1 str., 02-097 Warsaw, Poland.

[5]Department of Pharmaceutical Biology, Medical University of Warsaw, Banacha 1, Warsaw 02-097, Poland

[6] Microbiota Lab, Department of Pharmaceutical Microbiology and Bioanalysis, Medical University of Warsaw, Banacha 1, Warsaw 02-097, Poland

* Correspondence: joanna.kolmas@wum.edu.pl;

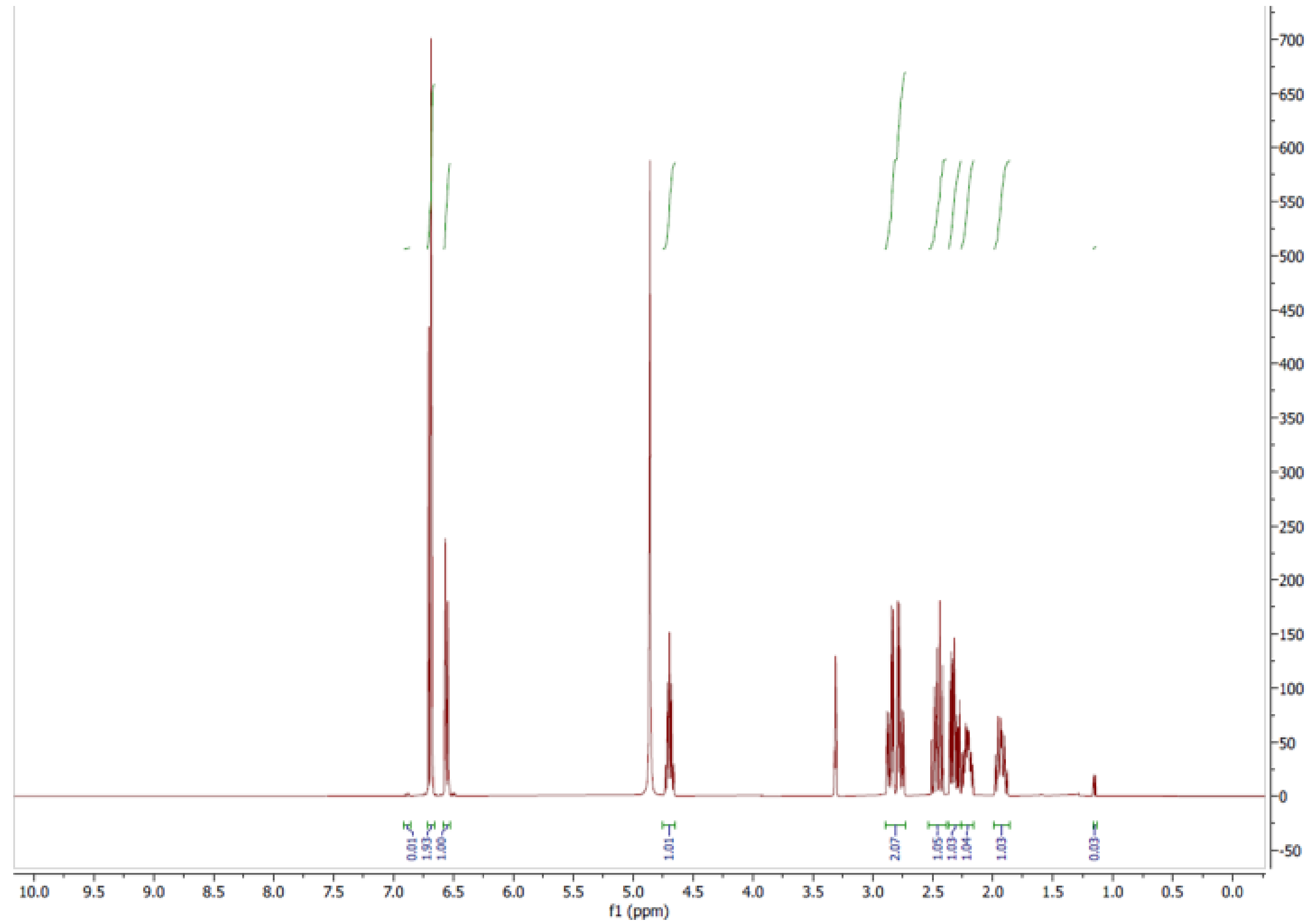

Figure S1. **^{1}H NMR of the sample of the DHPV (5-[(3,4-dihydroxyphenyl)methyl]oxolan-2-one) used in this study.** ^{1}H NMR (400 MHz, CD_3OD) δ 8.68 [br s, 2 x OH, ~0H (nominally 2H; exchanged into deuterium], 6.69 (br d, J = 10.6 Hz, 1H, C^{5}H) overlapping 6.69 (br s, 1H, C^{2}H), 6.55 (dd, J = 8.0, 2.1 Hz, 1H, C^{6}H), {4.74-4.66 (m, 1H, CHO; [4.70 (pseudo-p, J = 6.3 Hz, 1H)]}, 2.86 (dd, J = 14.0, 6.2 Hz, 1H), 2.77 (dd, J = 14.0, 6.1 Hz, 1H), 2.52-2.41 (m, 1H), 2.32 (ddd, J = 17.7, 9.4, 4.8 Hz, 1H), 2.26-2.17 (m, 1H), 1.99-1.88 (m, 1H)

Table S1. Validation data for the HPLC method.

<table>
<tr><th>Parameter</th><th>Acceptance criteria</th><th>Result</th></tr>
<tr><td colspan="2">Assay</td><td></td></tr>
<tr><td>Specificity</td><td>No significant interference of the API peak with possible peaks of the placebo and blank</td><td>A small peak with the retention time of the API peak was observed in the chromatogram of the placebo solution.
The area of this peak is approximately 0.05% of the API peak area at 100% of the nominal concentration - negligible/insignificant impact on the results of the determination of assay of active substance.
Furthermore, no presence / interference of additional peaks from the blank with the API peak were detected.</td></tr>
<tr><td>Linearity</td><td>Perform the linearity test on at least five concentration levels within the range: 50 - 150% of nominal concentration

Acceptance criteria:
- $R^2 \geq 0.995$
- Intercept ≤ 2.0% of the response at the concentration corresponding to 100% of the nominal concentration</td><td>- $R^2 = 1.000$
- Intercept = 1.5% of the response at the concentration corresponding to 100% of nominal concentration</td></tr>
<tr><td>Accuracy (recovery)</td><td>Perform the recovery test on three samples (placebo + API) on each of the three levels:
- 80%, 100%, and 120% of nominal concentration

Acceptance criteria:</td><td>
<table>
<tr><th>Concentration level, %</th><th>Sample</th><th>Theoretical amount of API, mg/ml</th><th>Calculated amount of API, mg/ml</th><th>Recovery, %</th><th>Average recovery, %</th><th>RSD, %</th></tr>
<tr><td rowspan="2">80%</td><td>1</td><td>0.35160</td><td>0.35330</td><td>100.48</td><td rowspan="2">101.3</td><td rowspan="2">0.7</td></tr>
<tr><td>2</td><td>0.34120</td><td>0.34669</td><td>101.61</td></tr>
</table>
</td></tr>
</table>

Parameter	Acceptance criteria	Result						
	Mean recovery at each of the three levels: within the range 98.0% - 102.0% of the theoretical value. RSD of recovery values at each of the three levels: ≤ 2.0%		3	0.33000	0.33589	101.78		
		100%	4	0.40120	0.40172	100.13	100.9	0.7
			5	0.42560	0.42994	101.02		
			6	0.40080	0.40732	101.63		
		120%	7	0.49280	0.50603	102.68	101.3	1.2
			8	0.48920	0.49069	100.31		
			9	0.50080	0.50590	101.02		
Related substances								
Specificity	No significant interference of the API peak with possible placebo and blank peaks. No significant interference of impurities peaks with possible blank, placebo, and API peaks.	A small peak with the retention time of the API peak was observed in the chromatogram of the placebo solution. The area of this peak is approximately 0.05% of the API peak area at 100% of the nominal concentration - negligible/insignificant impact on the results of the determination of the assay of active substance. Furthermore, no presence / interference of additional peaks from the placebo and blank with the API peak and impurities peaks were detected. No interference of the API peak with impurities peaks was detected.						
Linearity	For the main peak (API): Perform the linearity test on at least five concentration levels within the range: 0.05% (disregard level) - 150% of the nominal concentration of API in the sample solution. Acceptance criteria: - $R^2 \geq 0.995$	For the main peak (API): - $R^2 = 1.000$ - Intercept = 0.1% of the response at the concentration corresponding to 100% of nominal concentration. For unspecified impurities (as API): - $R^2 = 0.999$ - Intercept = -2.2% of the response at a concentration level 100% of the limit (limit=0.5%)						

<table>
<tr><th>Parameter</th><th>Acceptance criteria</th><th>Result</th></tr>
<tr><td></td><td>- Intercept ≤ 2.0% of the response for the concentration corresponding to 100% of the nominal concentration

<u>For unspecified impurities (as API):</u>
Perform the linearity test on at least five API concentration levels (used as the impurity marker) within the range: 0.05% (disregard level) - 150% of the limit (limit = 0.5%)

Acceptance criteria:
- $R^2 \geq 0.990$
- Intercept ≤ 5.0% of the response for the concentration corresponding to 100% of the limit (limit=0.5%)</td><td></td></tr>
<tr><td>Accuracy (recovery)</td><td>Perform the recovery of Unspecified impurity (as API) test on three samples of placebo spiked with API on each of five concentration levels within the range:
0.05% (disregard level) - 120% of the limit for Unspecified impurity (limit = 0.5%)

Mean recovery at each of the levels (respectively):
within the range 80% - 120% of the theoretical value.</td><td>
<table>
<tr><th>Concentration level, %</th><th>Sample</th><th>Theoretical amount of API, mg/ml</th><th>Calculated amount of API, mg/ml</th><th>Recovery, %</th><th>Average recovery, %</th><th>RSD, %</th></tr>
<tr><td rowspan="3">0.05 (disregard level)</td><td>1</td><td>0.00520625</td><td>0.0055663</td><td>106.9</td><td rowspan="3">102.5</td><td rowspan="3">4.1</td></tr>
<tr><td>2</td><td>0.00505625</td><td>0.0051588</td><td>102.0</td></tr>
<tr><td>3</td><td>0.00518125</td><td>0.0051050</td><td>98.5</td></tr>
<tr><td></td><td>4</td><td>0.02603125</td><td>0.0266320</td><td>102.3</td><td>97.7</td><td>4.5</td></tr>
</table>
</td></tr>
</table>

Parameter	Acceptance criteria	Result						
	RSD of recovery values at each of the levels: ≤ 10%	0.25 (50% of the Unspecified impurity limit (0.5%))	5	0.02528125	0.0245869	97.3		
			6	0.02590625	0.0242333	93.5		
		0.40 (80% of the Unspecified impurity limit (0.5%))	7	0.04165000	0.0397097	95.3	94.9	1.3
			8	0.04045000	0.0387717	95.9		
			9	0.04145000	0.0387409	93.5		
		0.50 (100% of the Unspecified impurity limit (0.5%))	10	0.05206250	0.0497274	95.5	95.2	2.5
			11	0.05056250	0.0492431	97.4		
			12	0.05181250	0.0479822	92.6		
		0.60 (120% of the Unspecified impurity limit (0.5%))	13	0.06247500	0.0591301	94.6	94.6	1.4
			14	0.06067500	0.0581537	95.8		
			15	0.06217500	0.0579769	93.2		

Figure S2 (a-d). Representative chromatograms from validated HPLC method.

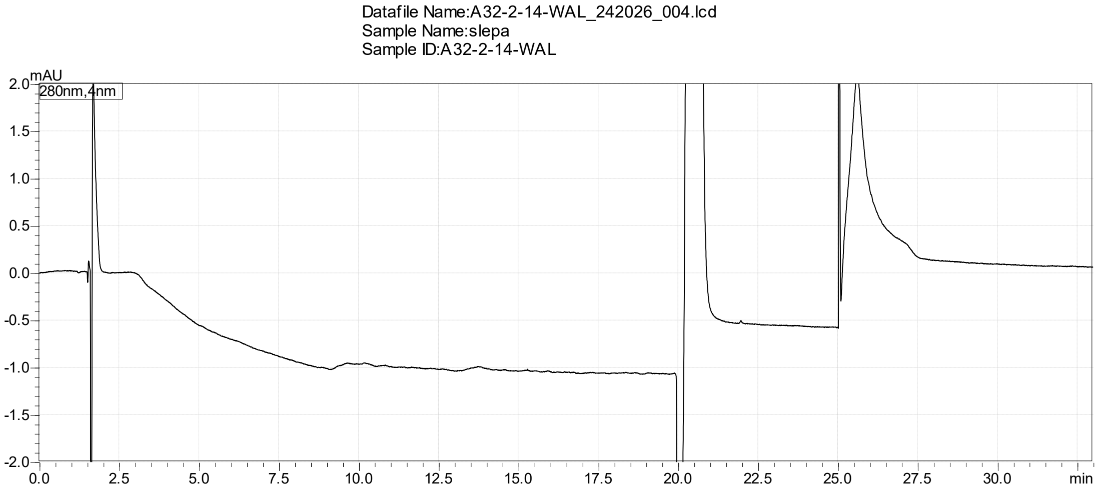

Figure S2a. The example of blank chromatogram

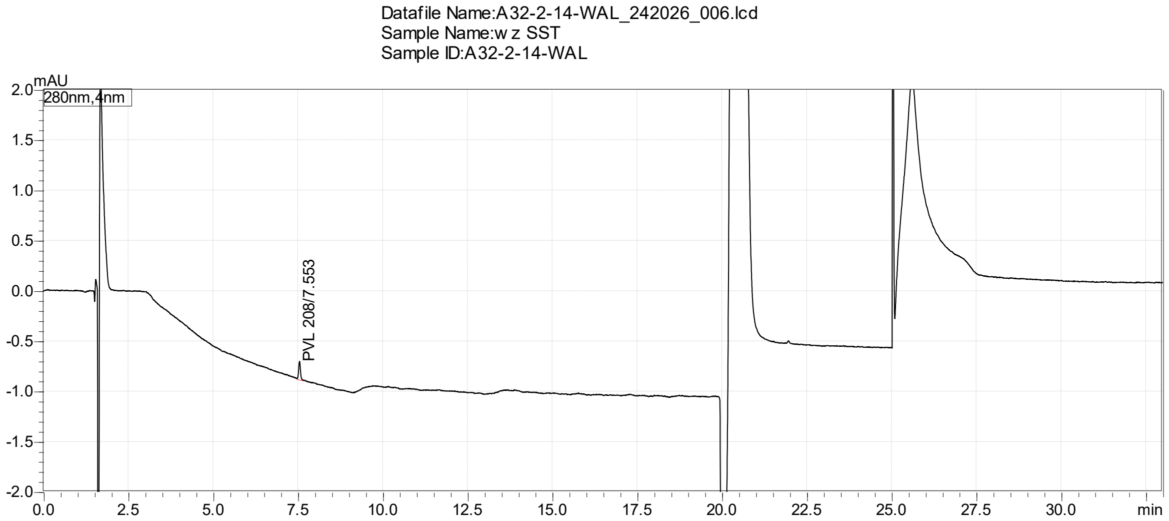

Figure S2b. The example of Sensitivity test solution – Related substances test chromatogram

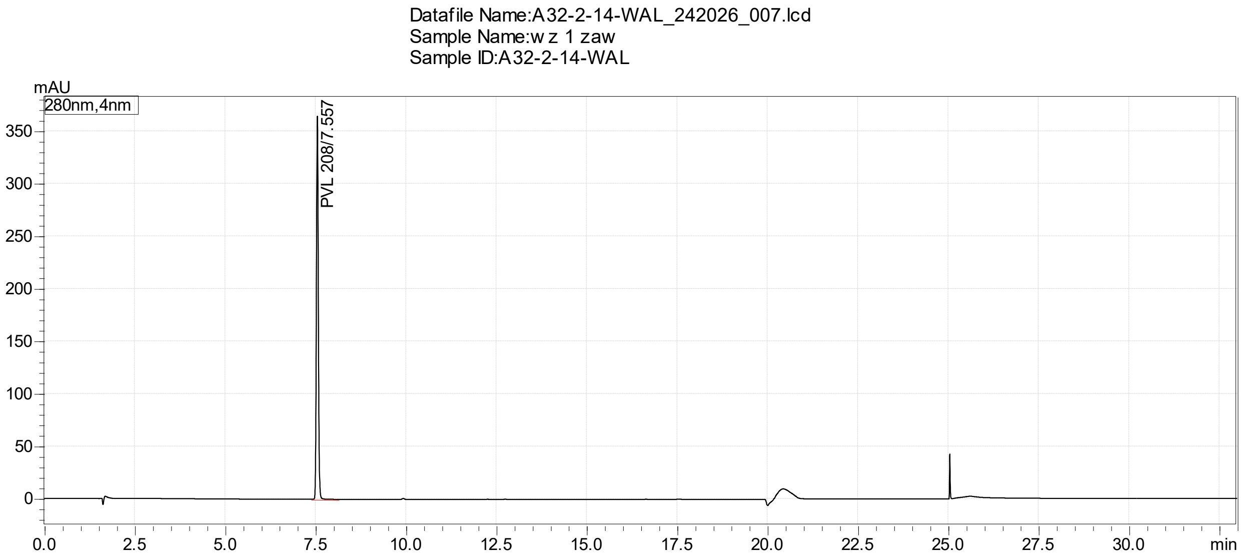

Fig. S2c. The example of Standard solution – Assay test chromatogram

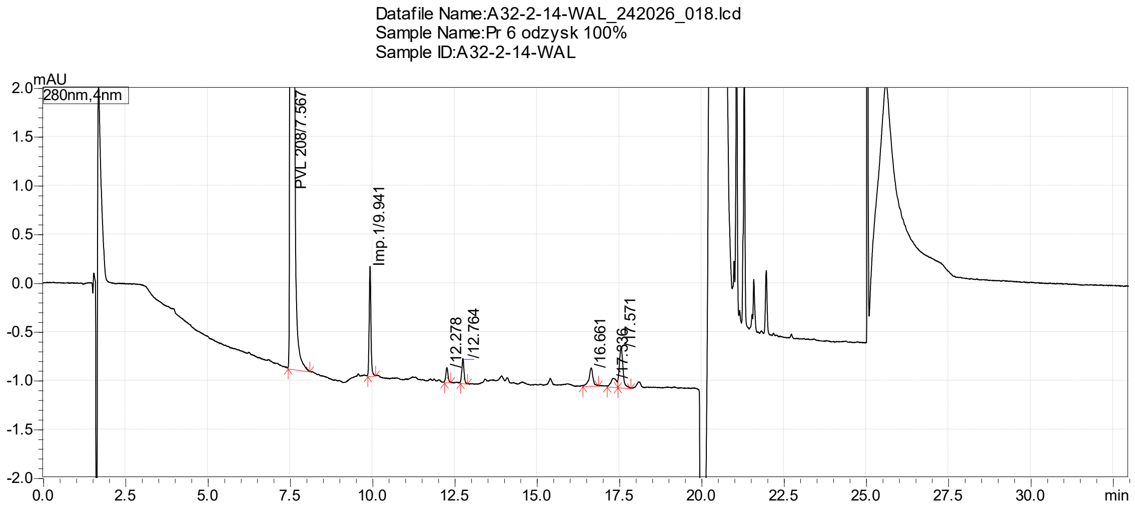

Fig. S2d. The example of sample solution chromatogram

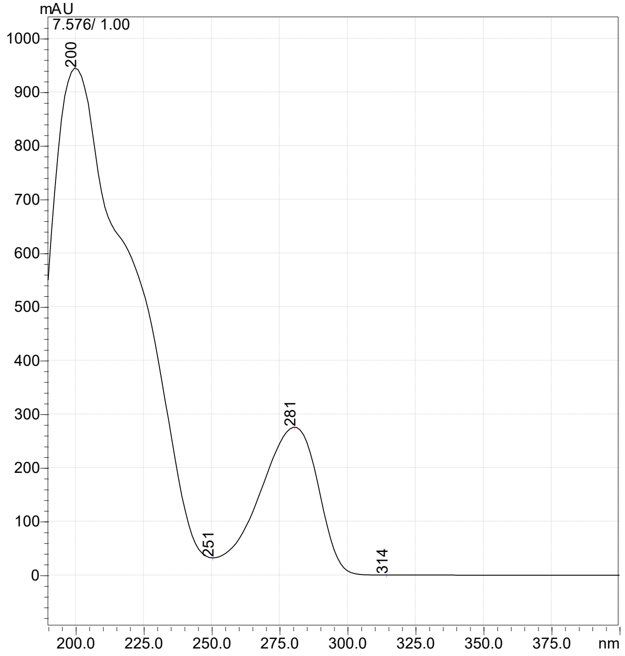

Figure S3. UV spectra of the DHPV (standard sample, concentration 0,4 mg/ml)

Figure S4. Main impurity (Impurity 1) ^{1}H NMR spectrum

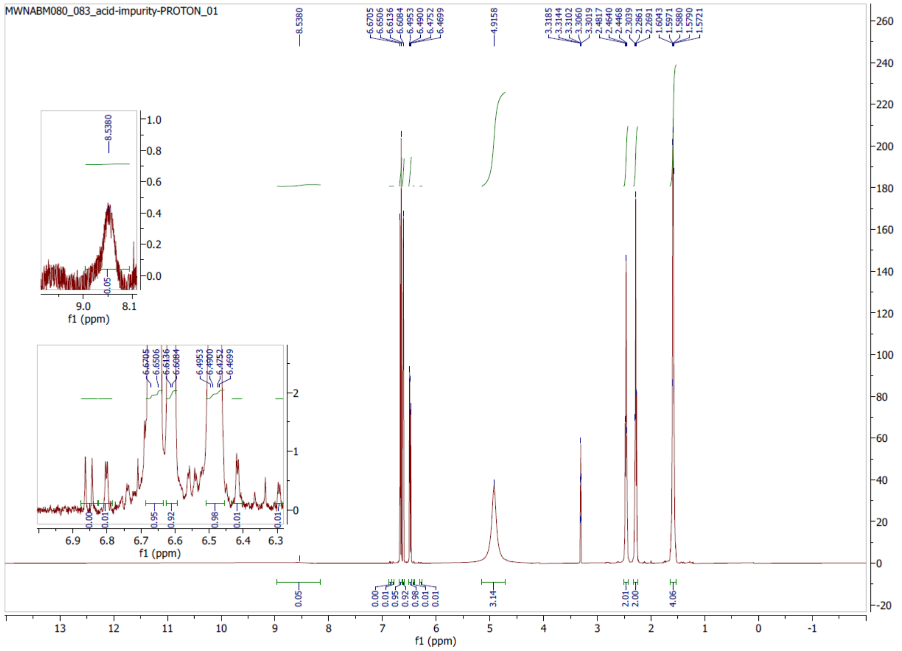

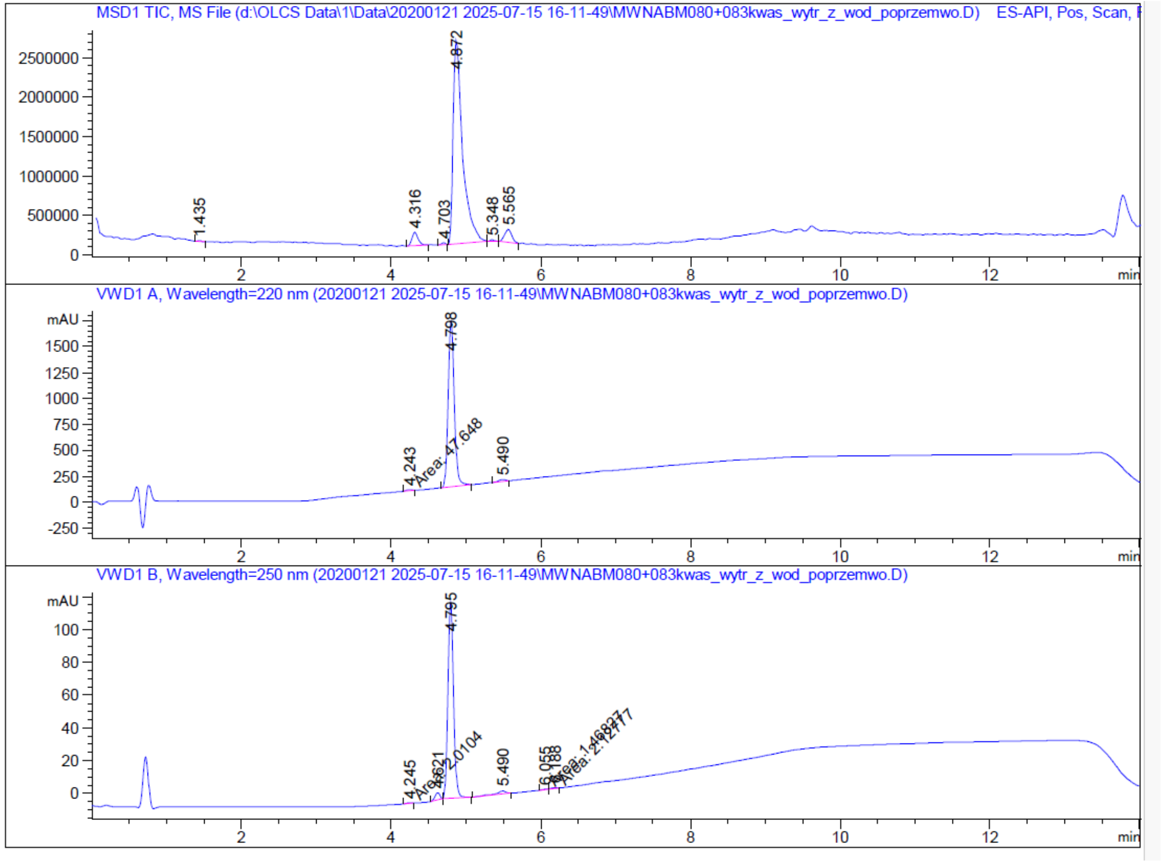

Figure S5. HPLC chromatogram of the Impurity 1.

Table S2. Compatibility of DHPV with selected excipients was evaluated under accelerated conditions (40 ± 2°C). Impurity profiles are expressed as percentage content (HPLC results).

Nr	Name	Time point	Conditions	Content of impurities [%]		
				**Imp. 1**	**Unspecified impurities**	**Total content of impurities**
API	DHPV	Initial	-	not tested		
		Day 14	40 ± 2˚C	0.185	0.045 (RRT=1.557)	0.23
		Day 28		0.201	0.046 (RRT=1.563)	0.25
1	DHPV + petrolatum (1:10)	Initial	-	not tested		
		Day 14	40 ± 2˚C	0.189	0.049 (RRT=1.559)	0.24
		Day 28		0.194	0.048 (RRT=1.562)	0.24
2	DHPV + medium-chain-triglycerides (1:10)	Initial	-	not tested		
		Day 14	40 ± 2˚C	0.194	0044 (RRT=1.558)<br>0.066 (RRT=4.084)*<br>0.062 (RRT=4.337)*	0.37**
		Day 28		0.188	0.050 (RRT=1.563)<br>0.114 (RRT=4.061)*<br>0.083 (RRT=4.301)*	0.44**
3	DHPV + paraffinum liquidum (1:10)	Initial	-	not tested		
		Day 14	40 ± 2˚C	0.207	0.048 (RRT=1.561)	0.26
		Day 28		0.195	0.049 (RRT=1.563)	0.24
4	DHPV + castor oil (1:10)	Initial	-	not tested		

Nr	Name	Time point	Conditions	Content of impurities [%]		
				Imp. 1	Unspecified impurities	Total content of impurities
		Day 14	40 ± 2°C	0.198	0.045 (RRT=1.560)	0.24
		Day 28		0.201	0.045 (RRT=1.562)<br>0.053 (RRT=2.652)	0.30
5	DHPV + 1,2-propanediol (1:5)	Initial	-	not tested		
		Day 14	40 ± 2°C	0.211	0.061 (RRT=1.526)<br>0.088 (RRT=1.561)<br>0.048 (RRT=4.361)<br>0.169 (RRT=4.468)	0.58
		Day 28		0.213	0.045 (RRT=1.032)<br>0.101 (RRT=1.528)<br>0.140 (RRT=1.563)<br>0.045 (RRT=1.871)<br>0.166 (RRT=4.340)	0.71
6	DHPV + 1,3-propanediol (1:5)	Initial	-	not tested		
		Day 14	40 ± 2°C	0.211	0.086 (RRT=1.525)<br>0.121 (RRT=1.561)	0.42
		Day 28		0.212	0.159 (RRT=1.528)<br>0.199 (RRT=1.563)	0.57
7	DHPV + polyethylene glycol 300 (1:5)	Initial	-	not tested		

Nr	Name	Time point	Conditions	Content of impurities [%]		
				Imp. 1	Unspecified impurities	Total content of impurities
		Day 14	40 ± 2˚C	0.218	0.053 (RRT=1.139)<br>0.057 (RRT=1.180)<br>0.051 (RRT=1.217)<br>0.061 (RRT=1.268)<br>0.103 (RRT=1.527)<br>0.145 (RRT=1.563)	0.69
		Day 28		0.227	0.061 (RRT=1.091)<br>0.089 (RRT=1.138)<br>0.097 (RRT=1.179)<br>0.093 (RRT=1.214)<br>0.066 (RRT=1.247)<br>0.098 (RRT=1.266)<br>0.152 (RRT=1.528)<br>0.198 (RRT=1.563)	1.1
8	DHPV + plant glycerine (1:5)	Initial	-	not tested		
		Day 14	40 ± 2˚C	0.206	0.156 (RRT=0.825)<br>0.051 (RRT=1.563)	0.41
		Day 28		0.201	0.319 (RRT=0.828)<br>0.051 (RRT=1.563)	0.57

Nr	Name	Time point	Conditions	Content of impurities [%]		
				Imp. 1	Unspecified impurities	Total content of impurities
9	DHPV + isopropyl myristate (1:1)	Initial	-	not tested		
		Day 14	40 ± 2°C	0.181	0.048 (RRT=1.564)	0.23
		Day 28		0.198	0.045 (RRT=1.563)<br>0.046 (RRT=1.873)	0.29
10	DHPV + cera alba (1:1)	Initial	-	not tested		
		Day 14	40 ± 2°C	0.199	0.047 (RRT=1.564)	0.25
		Day 28		0.213	0.046 (RRT=1.562)<br>0.049 (RRT=1.871)	0.31
11	DHPV + cetostearyl alcohol (1:1)	Initial	-	not tested		
		Day 14	40 ± 2°C	0.204	0.046 (RRT=1.565)	0.25
		Day 28		0.207	0.045 (RRT=1.562)	0.25
12	DHPV + cetyl palmitate (1:1)	Initial	-	not tested		
		Day 14	40 ± 2°C	0.203	0.044 (RRT=1.566)	0.25
		Day 28		0.213	0.047 (RRT=1.562)	0.26
13	DHPV + sorbitan monostearate (1:1)	Initial	-	not tested		
		Day 14	40 ± 2°C	0.185	0.043 (RRT=1.566)	0.23
		Day 28		0.180	<0.05	0.18
14	DHPV + polysorbate 60 (1:1)	Initial	-	not tested		
		Day 14	40 ± 2°C	0.203	0.048 (RRT=1.567)	0.25

Nr	Name	Time point	Conditions	Content of impurities [%]		
				Imp. 1	Unspecified impurities	Total content of impurities
		Day 28		0.198	0.050 (RRT=1.562)	0.25

Table S3. Representative data on the stability of formulations 1 and 2. (Packaging: aluminium tubes with membrane and screw cap (19 mm × 75 mm), approx. 8 g).

FORMULATION1 (0.5%)								
Conditions (°C / % RH)	Time point	External appearance	Microscopic analysis	DHPV content		Impurities content (%)		
				%	% declared content	Imp. 1	Uknown impurities	Total
	Initial	white, dense, homogenous	particles (140-20 µm long and 80-7 µm are observed.	0.49	98.0 (98.87 – top; 98.90 – middle; 96.15 – bottom)	0.29	0.05 (RRT = 1.532) 0.08 (RRT = 1.568) 0.10 (RRT = 1.835) 0.26 (RRT = 1.880)	0.78
25/60	Month 3	white, dense, homogenous	irregular, columnar particles (76-16 µm long and 48-7 µm wide), with a tendency to aggregate	0.49	98.7 (99.64 – top; 97.83 – bottom)	0.27	0.07 (RRT=1.532) 0.07 (RRT=1.567) 0.11 (RRT=1.824) 0.29 (RRT= 1.867)	0.81
30/65	Month 3	white, dense, homogenous	irregular, columnar particles (168-14 µm long and 49-7 µm wide), with a tendency to aggregate	0.50	99.3 (99.21 – top; 99.37 – bottom)	0.28	0.06 (RRT=1.532) 0.07 (RRT=1.567) 0.12 (RRT=1.824)	0.80

							0.27 (RRT= 1.868)	
40/75	Day 28	white, dense, homogenous	-	0.50	100.1 (99.40 – top; 100.85 – bottom)	0.30	0.06 (RRT = 1.542) 0.07 (RRT = 1.576) 0.11 (RRT = 1.834) 0.25 (RRT = 1.878)	0.77
	Month 3	white, dense, homogenous	irregular, columnar particles (79-20 µm long and 43-8 µm wide), with a tendency to aggregate	0.51	101.2 (102.12 – top; 100.22 – bottom)	0.29	0.06 (RRT=1.535) 0.07 (RRT=1.570) 0.11 (RRT=1.828) 0.26 (RRT= 1.872)	0.78
**FORMULATION 1 (5%)**								
Conditions (°C / % RH)	Time point	External appearance	Microscopic analysis	DHPV content		Impurities content. %		
				%	% declared content	Imp. 1	Uknown impurities	Total
	Initial	Dark cream, dense, homogenous	particles (113-10 µm long and 40-4 µm are observed.	5.00	100.0 (100.29 – top; 99.76 – middle; 100.07 – bottom)	0.29	0.05 (RRT = 1.531) 0.07 (RRT = 1.567) 0.10 (RRT = 1.834) 0.26 (RRT = 1.878)	0.77
25/60	Month 3	Dark cream, dense, homogenous	irregular, columnar particles (147-18 µm long and 79-10 µm wide), with a tendency to aggregate	5.01	100.2 (100.24 – top; 100.10 – bottom)	0.30	0.07 (RRT = 1.548) 0.08 (RRT = 1.583) 0.12 (RRT = 1.845) 0.30 (RRT = 1.890)	0.87
30/65	Month 3	Dark cream, dense, homogenous	irregular, columnar particles (106-16 µm long and 98-8 µm wide), with a tendency to aggregate	4.94	98.8 (97.99 – top; 99.57 –bottom)	0.30	0.07 (RRT = 1.546) 0.08 (RRT = 1.582) 0.12 (RRT = 1.843) 0.28 (RRT = 1.888)	0.85
40/75	Day 28	Dark cream, dense, homogenous	-	5.08	101.7	0.30	0.06 (RRT = 1.542) 0.08 (RRT = 1.577)	0.80

					(100.69 – top;<br>102.62 –<br>bottom)		0.11 (RRT = 1.835)<br>0.25 (RRT = 1.879)	
	Month 3	Dark cream, dense, homogenous	irregular, columnar particles (160-12 µm long and 81-4 µm wide), with a tendency to aggregate	5.08	101.6<br>(101.55 – top;<br>101.57 –<br>bottom)	0.28	0.07 (RRT = 1.546)<br>0.07 (RRT = 1.581)<br>0.11 (RRT = 1.842)<br>0.25 (RRT = 1.886)	0.78
**FORMULATION 2 (0.5%)**								
Conditions (°C / % RH)	Time point	External appearance	Microscopic analysis	DHPV content		Impurities content. %		
				%	% declared content	Imp. 1	Uknown impurities	Total
	Initial	Thick, oily, cream-colored	Particles (80-7 µm and 35-5 µm) are observed.	0.49	98.5<br>(98.42 – top;<br>98.33 – middle;<br>98.89 – bottom)	0.31	0.05 (RRT = 1.533)<br>0.08 (RRT = 1.569)<br>0.10 (RRT = 1.836)<br>0.26 (RRT = 1.881)	0.81
25/60	Day 28	Thick, oily, cream-colored	-	0.50	99.7<br>(99.50 – top;<br>99.86 – bottom)	0.32	0.06 (RRT = 1.531)<br>0.09 (RRT = 1.568)<br>0.10 (RRT = 1.843)<br>0.26 (RRT = 1.889)	0.83
	Month 3	Thick, oily, cream-colored	irregular, columnar particles (101-21 µm long and 73-7 µm wide), with a tendency to aggregate	0.50	99.6<br>(99.83 – top;<br>99.44 – bottom)	0.32	0.08 (RRT=1.544)<br>0.10 (RRT=1.578)<br>0.10 (RRT=1.837)<br>0.29 (RRT=1.881)	0.89
30/65	Day 28	Thick, oily, cream-colored	-	0.50	99.7<br>(100.39 – top;	0.31	0.07 (RRT = 1.533)	0.83

					98.96 – bottom)		0.09 (RRT = 1.570)<br>0.11 (RRT = 1.846)<br>0.26 (RRT = 1.892)	
	Month 3	Thick, oily, cream-colored	irregular, columnar particles (102-20 µm long and 40-13 µm wide), with a tendency to aggregate	0.50	99.6<br>(99.81 – top;<br>99.35 – bottom)	0.32	0.08 (RRT=1.543)<br>0.10 (RRT=1.578)<br>0.11 (RRT=1.836)<br>0.30 (RRT=1.880)	0.91
40/75	Day 28	Thick, oily, cream-colored	-	0.49	98.4<br>(96.82 – top;<br>100.07 – bottom)	0.31	0.07 (RRT = 1.535)<br>0.08 (RRT = 1.572)<br>0.10 (RRT = 1.848)<br>0.26 (RRT = 1.894)	0.82
	Month 3	Thick, oily, cream-colored	irregular, columnar particles (195-25 µm long and 61-20 µm wide), with a tendency to aggregate	0.49	98.7<br>(97.92 – top;<br>99.41 – bottom)	0.32	0.08 (RRT=1.544)<br>0.08 (RRT=1.578)<br>0.11 (RRT=1.837)<br>0.29 (RRT=1.881)	0.88
**FORMULATION 2 (5%)**								
Conditions (°C / % RH)	Time point	External appearance	Microscopic analysis	DHPV content		Impurities content. %		
				%	% declared content	Imp. 1	Uknown impurities	Total
	Initial	Thick, oily, dark cream-colored	Particles observed in the preparation ranged from 8 to 144 µm in length and 6 to 61 µm in width. A significant part of the	5.01	100.2<br>(101.91 – top;<br>98.10 – middle;<br>100.65 – bottom)	0.31	0.05 (RRT = 1.533)<br>0.08 (RRT = 1.569)	0.81

			observed particles were 40 to 100 µm in length.				0.10 (RRT = 1.836)<br>0.26 (RRT = 1.881)	
25/60	Day 28	Thick, oily, dark cream-colored	-	5.03	100.5<br>(99.88 – top; 101.12 – bottom)	0.31	0.05 (RRT = 1.533)<br>0.08 (RRT = 1.569)<br>0.10 (RRT = 1.836)<br>0.26 (RRT = 1.881)	0.80
	Month 3	Thick, oily, dark cream-colored	irregular/columnar particles with a tendency to aggregate. The particles were 93-11 µm long and 57-6 µm wide. Individual particles were >80 µm long.	5.03	100.7<br>(101.24 – top; 100.14 – bottom)	0.31	0.06 (RRT=1.544)<br>0.09 (RRT=1.579)<br>0.11 (RRT=1.838)<br>0.29 (RRT=1.882)	0.86
30/65	Day 28	Thick, oily, dark cream-colored	-	4.99	99.8<br>(99.74 – top; 99.93 – bottom)	0.31	0.05 (RRT = 1.533)<br>0.08 (RRT = 1.569)<br>0.10 (RRT = 1.836)<br>0.26 (RRT = 1.881)	0.80
	Month 3	Thick, oily, dark cream-colored	irregular/columnar particles (117-22 µm and 59-5 µm) with a tendency to aggregate	5.01	100.2<br>(99.69 – top; 101.72 – bottom)	0.32	0.07 (RRT=1.544)<br>0.10 (RRT=1.579)<br>0.12 (RRT=1.838)<br>0.30 (RRT=1.882)	0.92
40/75	Day 28	Thick, oily, dark cream-colored	-	4.96	99.3<br>(98.19 – top; 100.31 – bottom)	0.31	0.05 (RRT = 1.533)<br>0.08 (RRT = 1.569)	0.79

							0.10 (RRT = 1.836)<br>0.25 (RRT = 1.881)	
	Month 3	Thick, oily, dark cream-colored	irregular/columnar particles (120-24 µm and 56-5 µm) with a tendency to aggregate.	5.06	101.2 (99.79 – top; 102.54 – bottom)	0.31	0.06 (RRT=1.544)<br>0.09 (RRT=1.579)<br>0.12 (RRT=1.838)<br>0.29 (RRT=1.882)	0.85

Figure S6. The representatives images taken from under optical microscopy for the formulation 1 and 2.

	**X40**	**X200**
**Formulation 1, 0,5%**	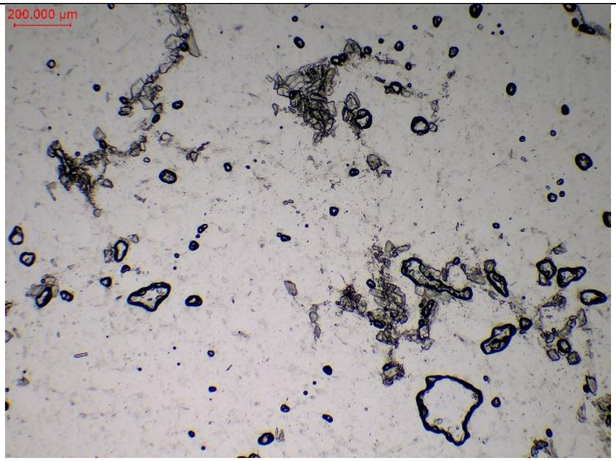	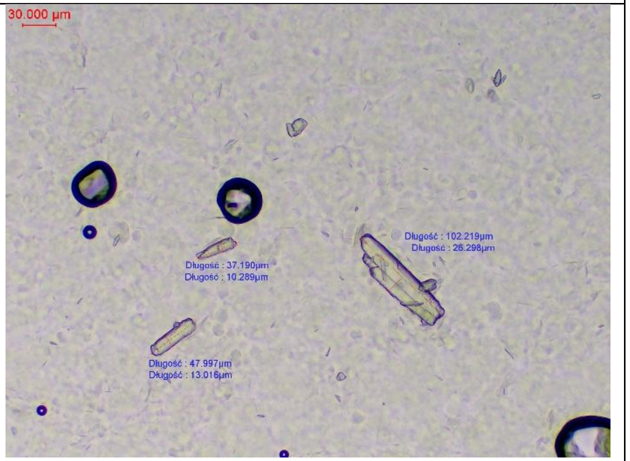

	X40	X200
**Formulation 1, 1%**	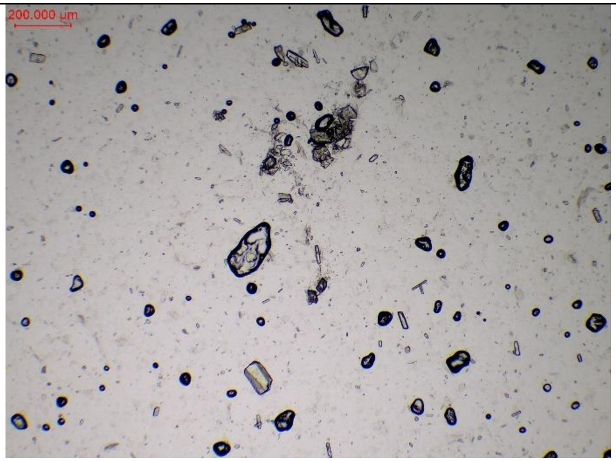	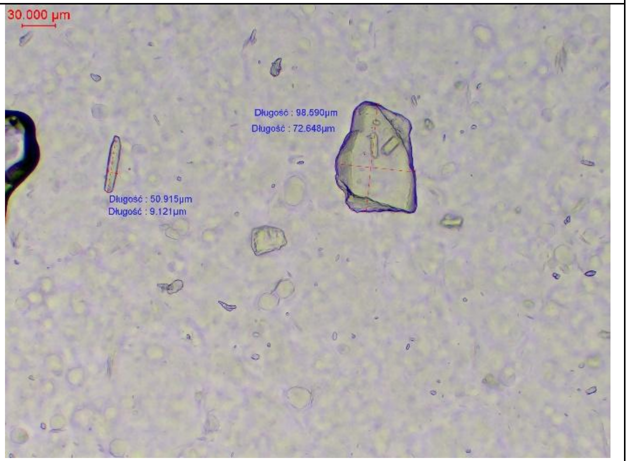

<table>
<tr><td></td><td>X40</td><td>X200</td></tr>
<tr><td>Formulation 1, 2%</td><td>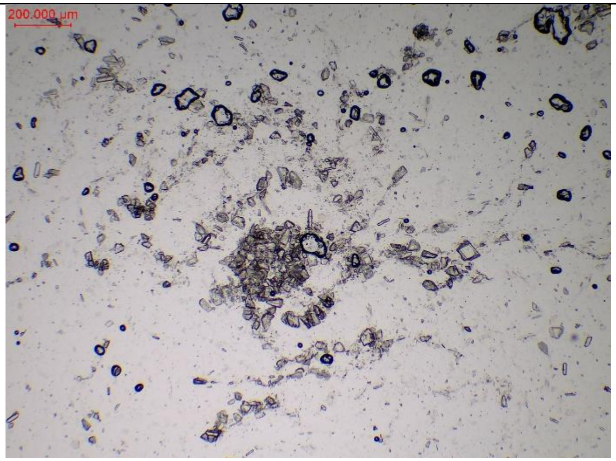
</td><td>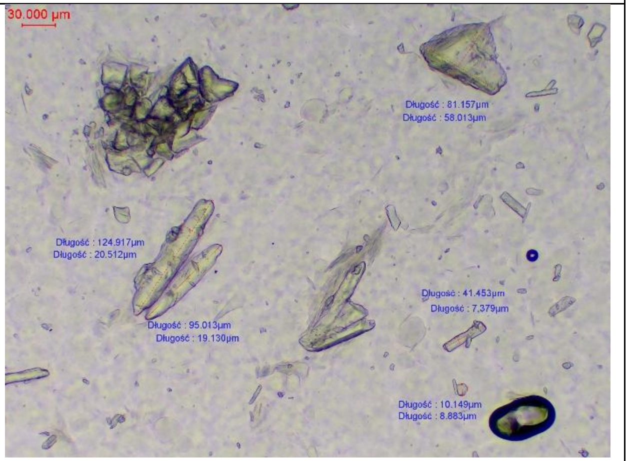
</td></tr>
<tr><td></td><td>X40</td><td>X200</td></tr>
<tr><td>Formulation 1, 5%</td><td>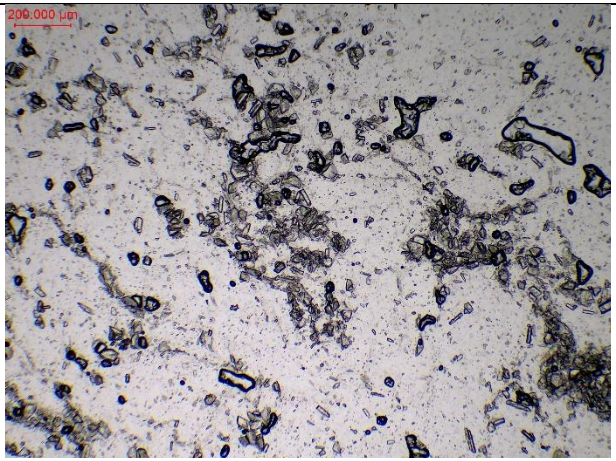
</td><td>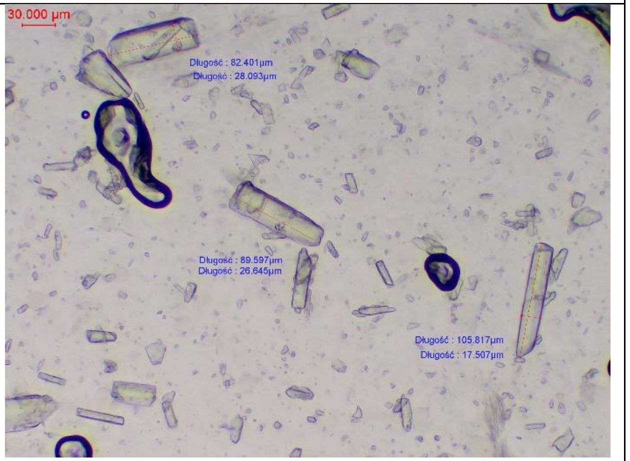
</td></tr>
</table>

<table>
<tr><td></td><td>X40</td><td>X200</td></tr>
<tr><td>Formulation 2, 0,5%</td><td>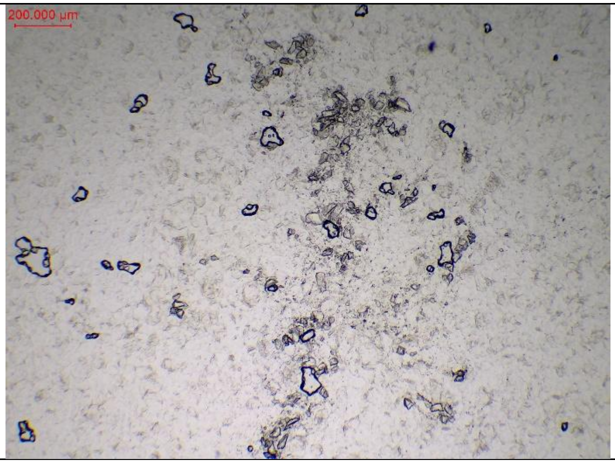
</td><td>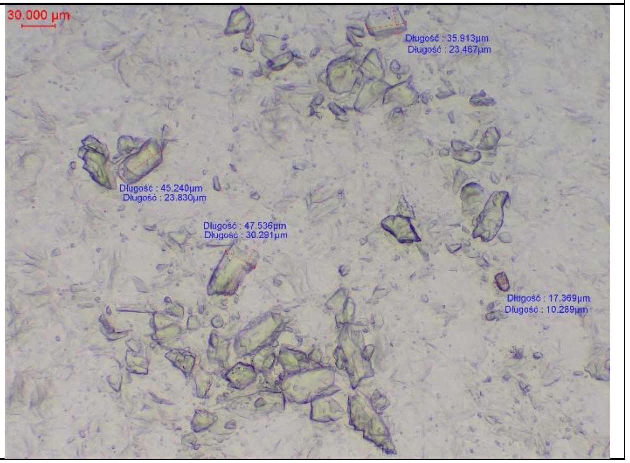
</td></tr>
</table>

<table>
<tr><td></td><td>X40</td><td>X200</td></tr>
<tr><td>Formulation 2, 1%</td><td>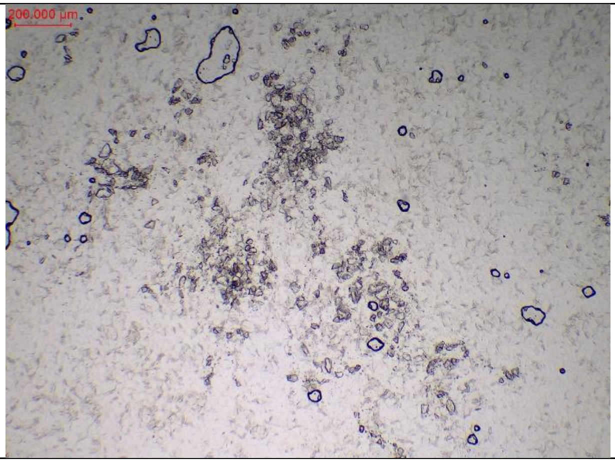
</td><td>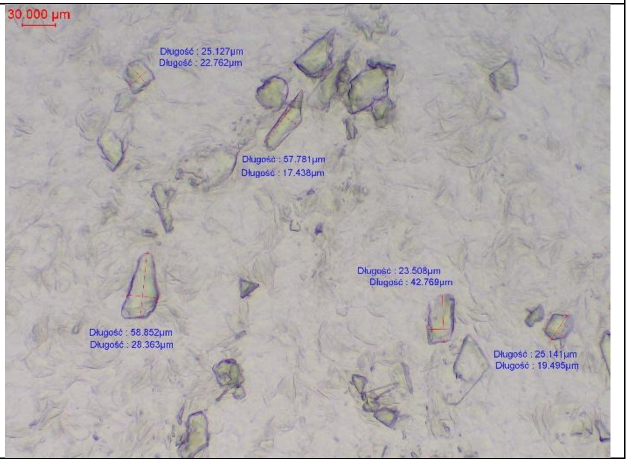
</td></tr>
</table>

<table>
<tr><td></td><td>X40</td><td>X200</td></tr>
<tr><td>Formulation 2, 2%</td><td>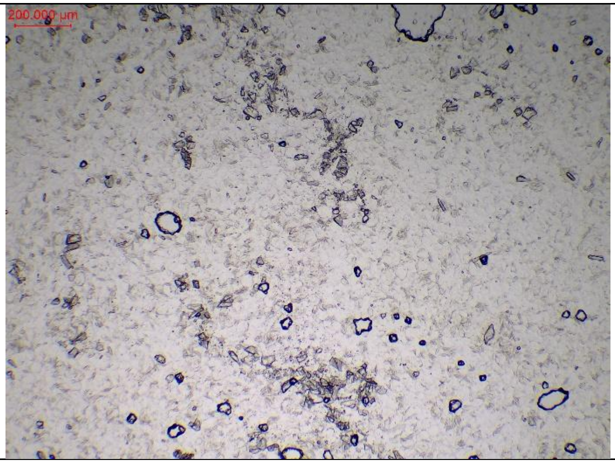</td><td>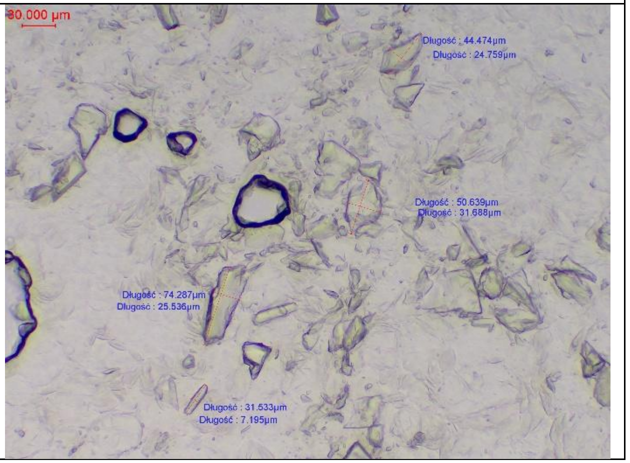
</td></tr>
</table>

<table>
<tr><td></td><td>X40</td><td>X200</td></tr>
<tr><td>Formulation 2, 5%</td><td>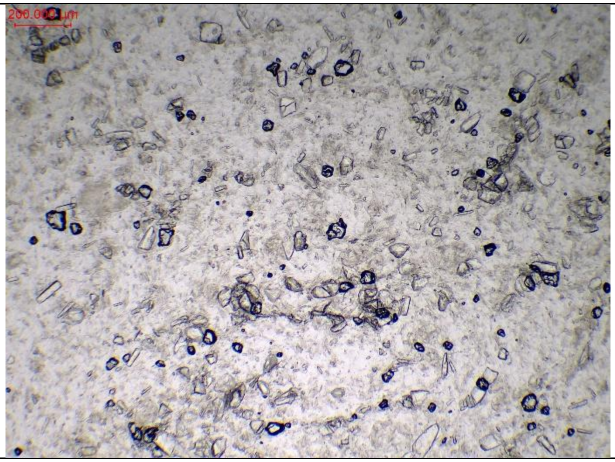</td><td>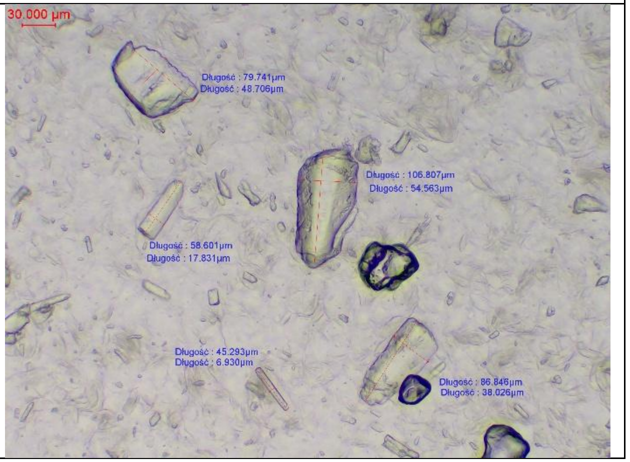
</td></tr>
</table>
